# Linking Individual Performance to Density‐Dependent Population Dynamics to Understand Temperature‐Mediated Genotype Coexistence

**DOI:** 10.1111/ele.70214

**Published:** 2025-09-15

**Authors:** Marjolein Bruijning, Luc De Meester, Marco D. Visser, Erlend I. F. Fossen, Héléne Vanvelk, Joost A. M. Raeymaekers, Lynn Govaert, Kristien I. Brans, Sigurd Einum, Eelke Jongejans

**Affiliations:** ^1^ Institute for Biodiversity and Ecosystem Dynamics University of Amsterdam Amsterdam the Netherlands; ^2^ Leibniz Institüt für Gewasserökologie Und Binnenfischerei (IGB) Berlin Germany; ^3^ Laboratory of Aquatic Ecology, Evolution and Conservation KU Leuven Leuven Belgium; ^4^ Institute of Biology Freie Universität Berlin Berlin Germany; ^5^ Institute of Environmental Sciences Leiden University Leiden the Netherlands; ^6^ CICERO Center for International Climate Research Oslo Norway; ^7^ Department of Biology, Centre for Biodiversity Dynamics, NTNU Norwegian University of Science and Technology Trondheim Norway; ^8^ Faculty of Biosciences and Aquaculture Nord University Bodø Norway; ^9^ Research Group Ecology, Evolution and Genetics (bDIV) Vrije Universiteit Brussel Brussels Belgium; ^10^ Radboud Institute for Biological and Environmental Sciences Radboud University Nijmegen the Netherlands; ^11^ Animal Ecology Netherlands Institute of Ecology Wageningen the Netherlands

**Keywords:** density‐dependence, integral projection modelling, intraspecific competition, modern coexistence theory, vital rates

## Abstract

The persistence of local populations exposed to climate change depends on their adaptive potential and on the ability of local individuals to compete with migrating conspecifics tracking environmental shifts. Modern coexistence theory (MCT) offers a framework for studying such competitive interactions among genotypes. However, MCT often focuses on emerging population‐level outcomes, aggregating over the underlying individual‐level interactions. We present a cross‐scale application of MCT, combining it with an Integral Projection Model (IPM), explicitly connecting individual performance to population‐level dynamics. We parameterise our model using experimental data on competing *Daphnia* genotypes from two latitudes. Consistent with observations, our model shows that higher temperatures increase the likelihood of competitive exclusion of Northern genotypes by Southern genotypes. Moreover, it reveals latitudinal variation in neonate sex ratios as a driver of temperature‐dependent evolutionary shifts. By identifying vital rates underlying population‐level competitive outcomes, our approach preserves the straightforward theoretical interpretability of MCT, while providing enhanced process‐level resolution through IPMs.

## Graphical abstract

We use a demographic model to explicitly connect individual performance to population‐level dynamics, parameterised using experimental data on competing Daphnia genotypes from two latitudes. We show that higher temperatures increase the likelihood of competitive exclusion of Northern genotypes by Southern genotypes, and highlight neonate sex ratios as an important driver of these temperature‐dependent competition outcomes.

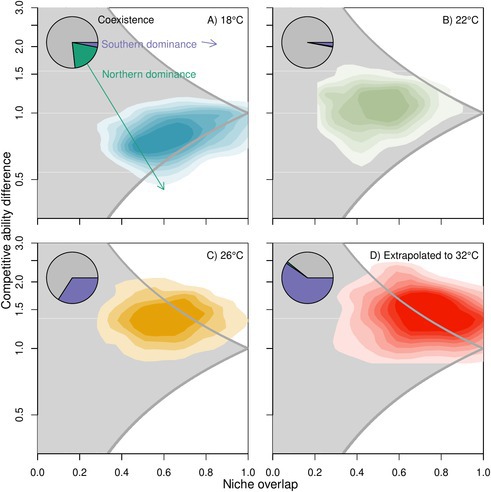

## 1 Introduction

Climate change profoundly impacts natural populations (Bellard et al. 2012; Parmesan 2006). The persistence of local populations hinges on their adaptive potential and on the ability of local individuals to compete with migrating individuals following environmental shifts (Alexander et al. 2015; Doorslaer et al. 2009; Hoffmann and Sgrò 2011; Parmesan 2006). Conspecific migrants better adapted to new conditions can outperform native genotypes, altering local gene pools (Penk et al. 2016). Yet, native and migrating conspecifics may also coexist, with one key mechanism for maintaining genetic polymorphism being negative frequency‐dependent selection (Ayala and Campbell 1974; Fitzpatrick et al. 2007; Olendorf et al. 2006; Pérez‐Tomé and Toro 1982). Here, rare genotypes have a fitness advantage, for example, due to more intense resource competition among genetically similar individuals (Fitzpatrick et al. 2007). As changes in local gene pools can have cascading effects on communities and ecosystems (Bellard et al. 2012), it is important to understand how climate change affects such conspecific competition (Amarasekare and Coutinho 2014).

Modern coexistence theory (MCT), often based on the foundational Lotka‐Volterra competition model, provides a framework to study competition (Broekman et al. 2019; Chesson 2000, 2018; Terry and Armitage 2024). While typically used to study different *species* (e.g., Adler et al. 2018), MCT can also describe competition between conspecific (asexual) *genotypes* (e.g., Letten et al. 2021). MCT's strength lies in its straightforward conceptual framework, coexistence outcomes being based on a simple theoretical criterion (Barabás et al. 2018) (Box 1). However, as MCT typically focuses exclusively on emerging population‐level dynamics, overlooking the fact that competitive interactions occur at the individual‐level, it provides limited mechanistic insights (Grainger et al. 2019; HilleRisLambers et al. 2012; Tilman 1987).

BOX 1 **Modern Coexistence Theory.**

In a two‐genotype Lotka‐Volterra competition model (LV‐model), changes in the abundance of each genotype (*N*_1_ and *N*_2_) can be written as:

(I) $$\frac{{\mathrm{dN}}_{1}}{\mathrm{dt}}=N_{1}r_{1}\left(1-\alpha_{11}N_{1}-\alpha_{12}N_{2}\right)$$

(II) $$\frac{{\mathrm{dN}}_{2}}{\mathrm{dt}}=N_{2}r_{2}\left(1-\alpha_{22}N_{2}-\alpha_{21}N_{1}\right)$$

(Broekman et al. 2019; Chesson 2018). Here, *r*_*1*_ and *r*_*2*_ are the intrinsic growth rates, $\alpha_{11}$ and $\alpha_{22}$ are the *intra*genotypic competition coefficients, and $\alpha_{12}$ and $\alpha_{21}$ are the *inter*genotypic competition coefficients, with $\alpha_{\mathrm{ij}}$ denoting the effect of genotype *j* on genotype *i*. This alternative formulation of the LV‐model expresses the α coefficients as per capita effects of each genotype on both their own and the other's per capita growth rate (Broekman et al. 2019; Chesson 2000; Vandermeer 1975). Under this formulation of the LV‐model, positive invasion growth rates at the other's equilibrium abundance are ensured when each genotype inhibits its own growth more than that of its competitor:

(III) $$\alpha_{11}>\alpha_{21}$$

and

(IV) $$\alpha_{22}>\alpha_{12}$$

This implies ‘mutual invasibility’, a necessary condition for stable coexistence (Grainger et al. 2019).

In modern coexistence theory, these conditions for coexistence are framed in terms of niche overlap and competitive ability difference. Niche overlap is defined as the ratio between interclonal and intraclonal competition:

(V) $$\rho =\sqrt{\frac{\alpha_{12}\alpha_{21}}{\alpha_{11}\alpha_{22}}}$$

The competitive ability difference between the two genotypes is calculated as:

(VI) $$k_{1}/k_{2}=\sqrt{\frac{\alpha_{11}\alpha_{12}}{\alpha_{22}\alpha_{21}}}$$

Following Chesson's (2018) framing, Equation (VI) does not incorporate differences in intrinsic growth rates (unlike e.g., Godoy and Levine 2014).

For stable coexistence, niche differences must be large enough to overcome differences in competitive ability, so that stable coexistence occurs when:

(VII) $$\rho <\frac{k_{1}}{k_{2}}<1/\rho$$

Rewriting Equation (VII) by expressing niche overlap and competitive ability differences in terms of the α coefficients results in the conditions for stable coexistence as shown in Equations (III) and (IV).

Population‐level dynamics naturally emerge from the performance of all individuals within their population. Individual performance results from the combined effects of individual vital rates, for example, development, survival and reproduction. Abiotic (e.g., temperature) and biotic (e.g., conspecific competition) factors can impact these vital rates differently: certain rates are more responsive, and effects in one stage may be compensated in another stage (Visser et al. 2016). Consequently, combined effects on population growth can partially or completely offset each other (Bruijning et al. 2022). Similarly, competitive interactions within and among genotypes can affect each vital rate with contrasting effects. In zooplankton, increasing population density decreases clutch sizes (Guisande 1993; Lampert 1978), but increases neonate body size (Guisande 1993). Temperature can either reduce or intensify competition (Milazzo et al. 2013; Tylianakis et al. 2008), impacting various vital rates simultaneously (Amarasekare and Coutinho 2014; Huxley et al. 2022; Lancaster et al. 2017; Laws and Belovsky 2010). A more mechanistic understanding of how the environment shapes coexistence, therefore, requires understanding how individual vital rates are impacted, and how these effects collectively propagate to the population level. While environmental‐mediated coexistence has been shown at the population level (Dudenhöffer et al. 2022; Terry 2025; Terry et al. 2021), little is known about how environmental change shapes intraspecific competition through each individual vital rate, and how their joint effects govern population‐level competitive outcomes (Amarasekare and Coutinho 2014).

There exists a handful of studies that have deciphered the importance of such underlying processes to coexistence outcomes (e.g., Adler et al. 2010; Chu and Adler 2015; Ellner et al. 2019; Lyu and Alexander 2023). For instance, combining MCT with demographic modelling, Chu and Adler (2015) highlight plant recruitment as the most important vital rate promoting coexistence across multiple grassland and shrubland communities, suggestive of general patterns of coexistence in plants. However, as of yet, these studies were limited to perennial plant species, reflecting the general trend that essentially all empirical studies applying MCT focus on primary producers (Buche et al. 2022).

To address this knowledge gap, we combine demographic modelling with MCT to quantify the contribution of underlying vital rates to (temperature‐dependent) coexistence outcomes of the primary consumer *Daphnia magna* . To this end, we use Integral Projection Models (IPMs), a type of population model that integrates across vital rates to describe population dynamics (Easterling et al. 2000; Merow et al. 2014). Our MCT‐IPM approach derives metrics analogous to those in MCT, framing coexistence outcomes in terms of niche overlap and competitive ability differences. Doing so, we can translate complex individual‐level dynamics to the conceptual simplicity that underlies MCT, benefiting from MCT's strong theoretical foundation, while also allowing us to quantify the contribution of underlying vital rates to (environmental‐dependent) coexistence outcomes.

To parameterise our model, we use laboratory data on competing *D. magna* genotypes. *D. magna* is a small crustacean, reproducing by cyclical parthenogenesis, and a keystone species in freshwaters. *Daphnia* genotypes can be locally adapted to temperature (Yampolsky et al. 2014), and competition for food, physical interactions, and infochemicals result in density‐dependent dynamics through various vital rates (Bruijning, tenBerge, and Jongejans 2018; Guisande 1993; Lampert 1978; Preuss et al. 2009). We monitored *D. magna* populations consisting of different genotypes collected at two latitudes, exposed to different temperatures. Previously, we showed that these genotypes differed in their responses to parasite infection (Bruijning et al. 2022); here, we focus on responses to temperature and its implications for coexistence. Parameterising our model with these data, we aim to answer how temperature mediates competition between genotypes from different latitudes, and which vital rates are instrumental for any temperature‐dependent coexistence.

## 2 Materials and Methods

### 2.1 Experimental Design and Measurements

**FIGURE 1:**
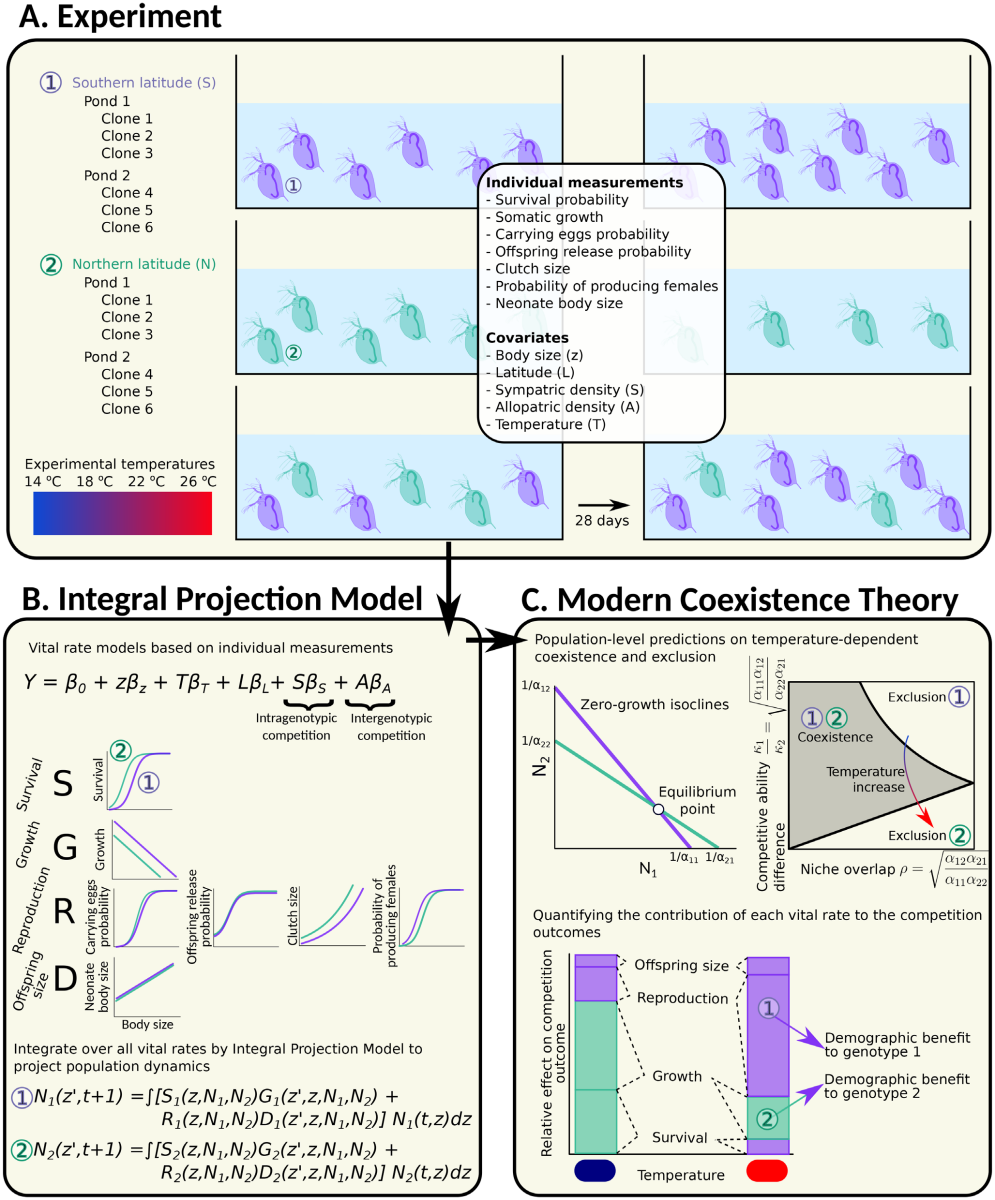
Conceptual overview of our experiments and modelling framework to study competitive interactions. (A) Overview of the experimental design, for which we used genotypes collected at two latitudes. We followed the dynamics of populations consisting of genotypes from a single latitude, or from both latitudes, at different temperatures. During 28 days, we performed measurements of various vital rates related to growth, reproduction, survival and offspring size on individuals within each population. (B) With these data, we fitted seven vital rate models. By adding sym‐ and allopatric abundances as covariates in each model, we estimated vital rate specific strength of intra‐ and intergenotypic competition. An IPM then integrated across all vital rates to describe changes in population size in discrete time. (C) This IPM was used to derive zero‐growth isoclines, that is, those population densities where population growth is predicted to be zero. The point where the isoclines for the two genotypes intersect, is the equilibrium point of the system. Population‐level intra‐ and intergenotypic competition coefficients can be derived from where these isoclines intersect with the *x*‐ and *y*‐axes. MCT frames these equilibrium points in terms of niche overlap and competitive ability difference (Box 1), in order to predict competition outcomes. By doing so for different environmental conditions (temperature in our case), we could quantify how the environment mediates competition outcomes. As the IPM is built from the underlying vital rates, we could also quantify the contribution of each of the underlying vital rates to competition outcomes. In the hypothetical example shown here, genotype‐specific survival and growth contribute positively to the dominance of genotype 2 in the blue environment, while reproduction and offspring size offer small advantages to genotype 1. In the red environment, somatic growth still offers an advantage to genotype 2, but this benefit is counterbalanced by advantages in survival, reproduction and offspring size for genotype 1.

To test for temperature‐mediated coexistence of *Daphnia* genotypes from different latitudes, we utilised data from a previously described experiment (Bruijning et al. 2022) (Figure 1A), in which we followed populations of single and competing genotypes. We concentrate on data from the first 4 weeks of the experiment, based on the stable population dynamics observed (Appendix 1) before a subsequent parasite‐infection phase described in Bruijning et al. (2022).

The experimental procedure involved the following steps: We used *D. magna* genotypes collected from two latitudes and two ponds at each latitude—in Belgium (South: 51°16′58.0″N 3°21′19.0″ E and 50°58′51.4″N 5°19′40.8″ E) and Norway (North, 63°26′56.6″N 10°27′11.0″ E and 63°35′16.1″N 10°43′46.0″ E). From each pond, we hatched ephippia in the lab and arbitrarily picked 3 genotypes, yielding 12 different genotypes in total. We designated the 6 Southern and 6 Northern genotypes as S_1‐6_ and N_1‐6_, respectively, with subscripts 1–3 and 4–6 indicating genotypes from the same pond. At the experimental start, we placed 12 female neonates in each aquaria (1.5 L), either as 12 individuals of a single genotype or 6 individuals of two different genotypes. Each of the 12 genotypes was used for a single‐genotype treatment at each of four temperatures (14°C, 18°C, 22°C and 26°C; resulting in 48 populations). Further, each of the 66 unique pairwise combinations (consisting of 36 inter‐latitudinal and 30 intra‐latitudinal combinations) was used once for competition treatments, stratified randomly across the highest three temperatures. In total, this resulted in 48 + 66 = 114 aquaria. Food was provided daily and aquaria were cleaned twice per week. We allowed populations to develop without culling for 28 days.

We monitored individuals and estimated population abundance (referred to as ‘density’) twice per week using R‐package *trackdem* (Bruijning, Visser, et al. 2018). Individual measurements were done by temporarily isolating two individuals per aquarium in permeable tubes for 3–4 days. This allowed us to monitor individuals while keeping them within their population, subjecting them to density‐dependent processes. We collected individual‐level data on survival, body size, and, if any, the number of eggs carried, number of released offspring, and offspring body size and sex. Starting from Day 14, isolated individuals were (destructively) sampled to identify their genotype using microsatellites (details in Bruijning et al. 2022).

### 2.2 Temperature‐Dependent Competition Outcomes

To describe how temperature shaped competition outcomes, we analysed the observed genotype identities from isolated individuals. We included identified genotypes from all 36 aquaria with Southern‐Northern genotype combinations (data sample size *n* = 241). We fitted Bayesian generalised linear mixed models to quantify the frequency of Southern genotypes in a population over time, accounting for a random effect of aquarium. We refer to this model as the ‘Observed genotype frequencies’:

(1) $$g\left(y_{\mathrm{ij}}\right)=d_{\mathrm{ij}}\left(\beta_{1}+u_{j}\right)+d_{\mathrm{ij}}T_{j}\beta_{2}+\epsilon_{\mathrm{ij}}$$

Here, *g()* is a logit link function giving the probability of sampling a Southern genotype, with the Northern genotype probability given by its complement $1-g\left(y_{\mathrm{ij}}\right)$. Subscripts denote individual *i* in aquarium *j*, *d* indicates day, *T* indicates standardised temperature. Estimated coefficients are given by $\beta_{1}$ and $\beta_{2}$; $u_{j}$ is the estimated random effect for aquarium *j* (corresponding to specific across‐latitude clonal combinations); and $\epsilon_{\mathrm{ij}}$ are residuals. We did not fit an intercept as at Day 0 (start of the experiment) the probability of sampling each genotype was equal (0.5).

### 2.3 Vital Rates

In all 144 aquaria, we measured seven individual‐level vital rates: survival probability (*n* = 1115), somatic growth (*n* = 1092), carrying eggs probability (*n* = 1073), neonate release probability at the next census conditional on having eggs (*n* = 352), clutch size (*n* = 306), probability of producing females conditional on producing offspring (*n* = 305), and neonate body size (*n* = 305) (details in Bruijning et al. (2022)).

We used these measurements to fit vital rate models, using Bayesian generalised linear mixed models. We included body size (continuous, standardised), temperature (continuous, standardised), and sympatric and allopatric densities (continuous) as covariates, along with latitude as a two‐level factor. Sympatric and allopatric densities were estimated by multiplying total population density by their predicted frequencies from the fitted genotype frequency model (Equation 1). As we were interested in competitive interactions *between* latitudes, sympatric and allopatric densities were defined at the latitude level. We, therefore, assume that genotypes compete equally with other genotypes from the same latitude. Note that in single‐latitude treatments, allopatric densities were set at 0. In the survival and somatic growth models, we additionally included sex as a two‐level factor (males were not included in reproduction‐related vital rates). Models also included a random intercept for clonal identity (12 levels). Depending on the vital rate, we used different regression models: logistic regression for binomial data (survival, carrying eggs probability, offspring release probability, female probability), Poisson regression for count data (clutch size) and linear regression for continuous data (somatic growth and neonate size).

For each vital rate, we compared five regression models, moving from simple to complex: starting with only 7 additive fixed effects (or 8, when including sex), to a model including 14 (or 15) fixed effects with three‐way interactions between latitude, temperature, and sym‐ or allopatric densities (Appendix 2.1). Models were fit in R 4.5.0 using the package *brms* (version 2.22.0) (Bürkner 2017), using four parallel chains and a thinning interval of 10 to reduce the size of the fitting objects. For clutch size, we used 100,000 iterations (with 50,000 burn‐in steps); for all other vital rates, we used 50,000 iterations (with 25,000 burn‐in steps). We checked convergence by ensuring Rhat < 1.01. We selected the best model based on leave‐one‐out cross‐validation across all draws and calculated the expected log pointwise predictive density (ELPD) to measure model performance. We used the best (i.e., highest ELPD) models to parameterise an Integral Projection Model (IPM).

### 2.4 Integral Projection Model

To link individual performance to population dynamics, we integrated across vital rates models using IPMs (Figure 1B). IPMs describe the discrete‐time dynamics of a population in which individuals are characterised by a continuous state variable (Easterling et al. 2000; Ellner et al. 2016; Ellner and Rees 2006; Merow et al. 2014). We created an IPM that tracks females only, as males did not contribute to population growth in our experiments (any sexual eggs were removed from the aquaria). Standardised body size (*z*) was the continuous variable, and we included standardised temperature (*T*), sym‐ and allopatric population density (*S* and *A*, respectively), latitude (*L*) and genotype (*C*) as covariates. Let *n* be the population size:
(2)

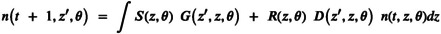




Equation (2) describes the body size distribution *z*' at time *t* + 1, given the body size distribution *z* at time *t*, for parameter set θ (i.e., combination of *T*, *S*, *A*, *L*, *C*).

The female survival probability is given by $S\left(z,\theta \right)$, estimating survival probabilities after a measurement interval of, on average, 3.5 days. To convert these to daily survival probabilities, we took the 3.5th root. Female somatic growth is given by probability density function $G\left(z^{\prime },z,\theta \right)$, describing the size distributions at *t* + 1 of female survivors, where:

(3) $$G\left(z^{\prime },z,\theta \right)\sim \text{Normal}\left(g\left(z,\theta \right),\sigma_{g}^{2}\right)$$

$g\left(z,\theta \right)$ describes the expected growth in millimetres; $\sigma_{g}^{2}$ is the variance given by the residual variance of the fitted growth model. Daily somatic growth rates were obtained by dividing observed growth by the census interval (3 or 4 days) prior to fitting models.

Reproduction $R\left(z,\theta \right)$ equals the product of four vital rates, so that: $R\left(z,\theta \right)=p_{e}\left(z,\theta \right)\;p_{r}\left(z,\theta \right)F\left(z,\theta \right)p_{f}\left(z,\theta \right)$. Here, $p_{e}\left(z,\theta \right)$ is the carrying eggs probability at the start of an interval, $p_{r}\left(z,\theta \right)$ is probability of releasing neonates 3–4 days later conditional on carrying eggs at the start, $F\left(z,\theta \right)$ is clutch size, and $p_{f}\left(z,\theta \right)$ is the probability of producing females. Division by 3.5 of the full reproduction kernel obtains daily reproduction estimates.

Finally, $D\left(z^{\prime },z,\theta \right)$ is a probability density function describing the neonates body size distribution, based on the expected offspring size at birth $\left(\phi \left(z,\theta \right)\right)$, and the variance around the expectation, calculated as the residual variance of the fitted offspring size model ($\sigma_{\phi }^{2}$).

(4) $$D\left(z^{\prime },z,\theta \right)\sim \text{Normal}\left(\phi \left(z,\theta \right),\sigma_{\phi }^{2}\right)$$

While the average census interval in our experiment was 3.5 days, by transforming each of the kernels as explained, our IPM projects daily dynamics. In Bruijning, tenBerge, and Jongejans (2018), we showed that results are qualitatively robust to the details of how daily kernels are obtained. The IPM was discretised into a 100 × 100 matrix (i.e., 100 size classes), with (standardised) body size ranging between −2.5 and 2.5 (corresponding to 0.3 and 4 mm, respectively). To avoid eviction, predicted transitions lower or higher than the size limits are added to the first or last size class, respectively (Williams et al. 2012).

### 2.5 Obtaining the Strength of Sympatric and Allopatric Competition

The discretized IPM projects asymptotic daily population growth rates (λ), calculated as the dominant eigenvalue of the constructed matrix. For each temperature, we calculated λ for different combinations of Northern and Southern densities (for the average genotype, that is, setting random genotype effects 0), ranging between 1 and 1000 individuals. We numerically derived zero‐growth isoclines, represented by the combinations of Northern and Southern densities that lead to zero population growth ($\log\;\left(\lambda \right)=0$), for each latitude. The intersection of the zero‐growth isoclines of the Northern and Southern genotypes represents the system's equilibrium point (Figure 1C). These isoclines essentially show an invasion analysis, corresponding to standard MCT definitions of mutual invasibility (Grainger et al. 2019). A linear model was used to determine where the isoclines intersected with the *x*‐ and *y*‐axes, providing intra‐ and intergenotypic competition coefficients (Figure 1C), used to approximate niche overlap and competitive ability difference (Box 1). We repeated these analyses by taking 1000 draws from the posterior distributions of each vital rate model, in order to estimate uncertainty in coexistence outcomes (note that we did not take into account uncertainty in our estimates of *S* and *A*, coming from uncertainty in our ‘Observed genotype frequencies’ model). For each draw, we recalculated zero‐growth isoclines, competition coefficients, niche overlap and competitive ability difference. In addition, we used the IPMs directly to simulate the dynamics of competing genotypes, requiring no assumptions on isocline linearity. Simulations started with six juveniles per latitude, matching observed neonate size distributions. We projected dynamics over 100 days, updating latitude‐ and density‐specific IPMs at each timestep, to reach equilibrium.

We then quantified the contribution of each vital rate to coexistence outcomes (Figure 1C). Here, we repeated the above analysis, substituting each vital rate of the Northern genotypes, one by one, with the corresponding vital rate function from the Southern genotypes, and *vice versa*. For each of these hypothetical scenarios, we again approximated the zero‐growth isoclines and intra‐ and intergenotypic competition coefficients, and calculated equilibrium proportions, niche overlap, and competitive ability differences. By taking 1000 draws from the posterior distributions of each vital rate, we obtained uncertainty estimates. Finally, we estimated variation in competitive outcomes among clonal pairs (details and results in Appendix 3).

## 3 Results

We described how we fitted an Integral Projection Model to experimental data on competing *Daphnia* genotypes from two latitudes, enabled by individual‐level data on individuals within their population. We then linked these IPM outcomes to MCT, deriving niche overlap and competitive ability difference. This allows us to assess how temperature mediates coexistence outcomes of Northern and Southern genotypes, as well as the underlying vital rates driving these competition outcomes.

### 3.1 Observed Genotype Frequencies

**FIGURE 2:**
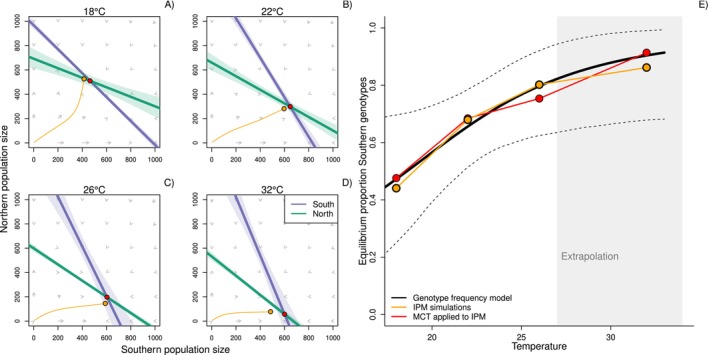
Applying modern coexistence theory to an Integral Projection Modelling framework. (A–D) Vector field plot showing population growth rates for different combinations of Southern (*x*‐axis) and Northern (*y*‐axis) densities, as projected by an IPM, and for different temperatures. Here, per vital rate, we averaged posterior draws from the posterior predictive distribution. Lines show the fitted null‐growth isoclines of Northern (teal) and Southern (purple) genotypes (shading indicates 95% confidence intervals of the mean). Circles show where the two lines intersect. (E) The obtained equilibrium densities translate into a predicted increasing equilibrium proportion of Southern genotypes with temperature (red line). The black line shows the observed proportion of Southern genotypes at the end of the experiment (Day 29), based on the fitted Genotype frequency model (see Equation 1). Here, dotted lines show the 2.5%–97.5% range of the predictive posterior distribution of the Genotype frequency model.

During the experiment, the frequency of Southern genotypes increased from parity with Northern genotypes to dominance in warmer conditions, indicating significant temperature‐dependent competition (*β*_2_ = 0.03; 95% CI [0.002, 0.05]). As a consequence, after 28 days, the proportions of Southern genotypes remained ~50% at 18°C but increased to ~80% at 26°C (black line in Figure 2B, details in Appendix 4). Except for populations at the lowest temperature (14°C), populations approached equilibrium densities during the experiment (Appendix 1).

### 3.2 Vital Rates

We found significant negative effects of sympatric density in four (i.e., somatic growth, carrying eggs probability, clutch size and neonate female probability) of the seven vital rates, implying competition between genotypes from the same latitude ($\beta_{S}$ values in Appendix 2.3). For somatic growth and clutch size, we additionally found negative effects of allopatric density ($\beta_{A}$), implying competition among genotypes from different latitudes. Neonate size, on the other hand, increased with both sym‐ and allopatric density. Temperature increased neonate release probabilities and clutch size ($\beta_{T}$) and intensified competition, reflected by negative interactions between temperature and sym‐ and allopatric density in various rates related to reproduction ($\beta_{\mathrm{TS}},\beta_{\mathrm{TA}}$). Northern genotypes experienced weaker allopatric density effects than Southern genotypes in terms of both neonate release and female probabilities ($\beta_{\mathrm{LA}}$). However, for the latter, this effect diminished at higher temperatures (significant three‐way interaction between latitude, density and temperature) ($\beta_{\mathrm{TLA}}$). We note that there was considerable uncertainty in model selection for most of the vital rates; we refer to Appendix 2 for the model selection, averaged coefficients, posterior distributions and a visual representation of the findings, raw data and residual trends.

### 3.3 Temperature‐Dependent Coexistence

Using our constructed IPM that integrates the temperature, density, and genotype effects across vital rates, we predict that the average predicted equilibrium proportion of Southern genotypes increases with temperature. We obtained these predictions both when based directly on IPM simulations of competing genotypes (Figure 2, orange colours) as well as on an application of MCT to our IPM (Figure 2, red colours), which approximates the competition coefficients using a linear model (teal and purple lines in Figure 2A–D). These results match the trends predicted from data on identified genotypes (black line in Figure 2E).

**FIGURE 3:**
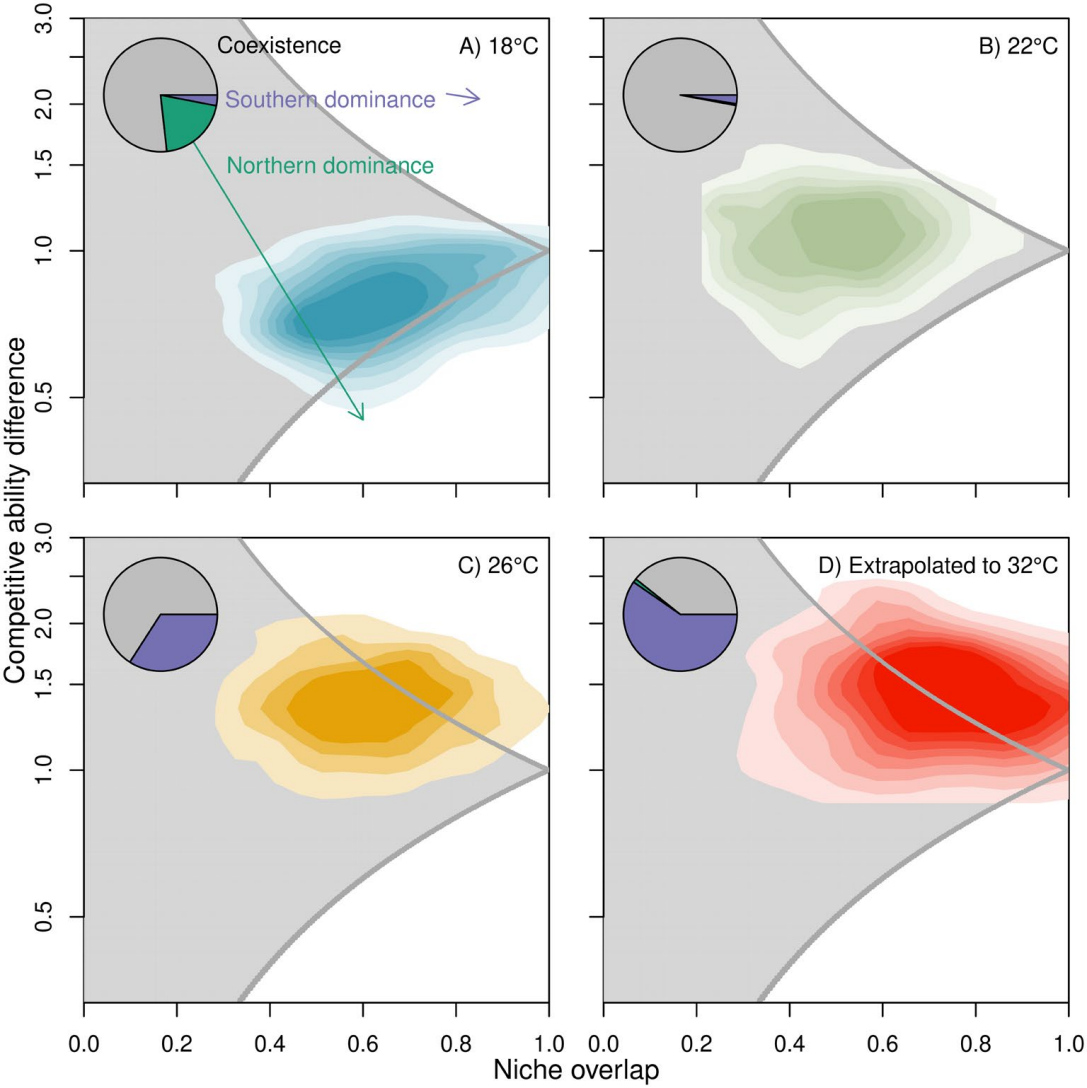
Temperature shifts niche overlap and competitive ability difference between Northern and Southern genotypes, changing competition outcomes. Graphical illustration of regions of coexistence as a function of niche overlap and competitive ability difference, at different temperatures (A: 18°C; B: 22°C; C: 26°C; D: 32°C). Coloured contour plots show variation due to parameter uncertainty in each of the underlying vital rates, not including random effects of genotypes within latitude. Pie charts in each panel summarise coexistence outcomes per temperature (purple: Southern dominance; teal: Northern dominance; grey: Stable coexistence).

Competition coefficients extracted from the approximated zero‐growth isoclines (Figure 2A–D) while incorporating parameter uncertainty in the underlying vital rates show temperature‐dependent shifts in niche overlap and competitive ability (Figure 3). At 18°C, the lowest temperature in the competition experiments, we predict conditions consistent with the requirements for stable coexistence in ~75% of the posterior draws, with exclusion of Southern genotypes predicted in most remaining cases (pie chart in Figure 3A). This pattern shows a systematic shift with temperature: when extrapolating to 32°C (26°C was the highest experimental temperature), the MCT‐IPMs predict essentially zero exclusion of Southern genotypes, and exclusion of Northern genotypes in ~60% of the posterior draws (Figure 3D).

### 3.4 Vital Rate Decomposition Analysis

**FIGURE 4:**
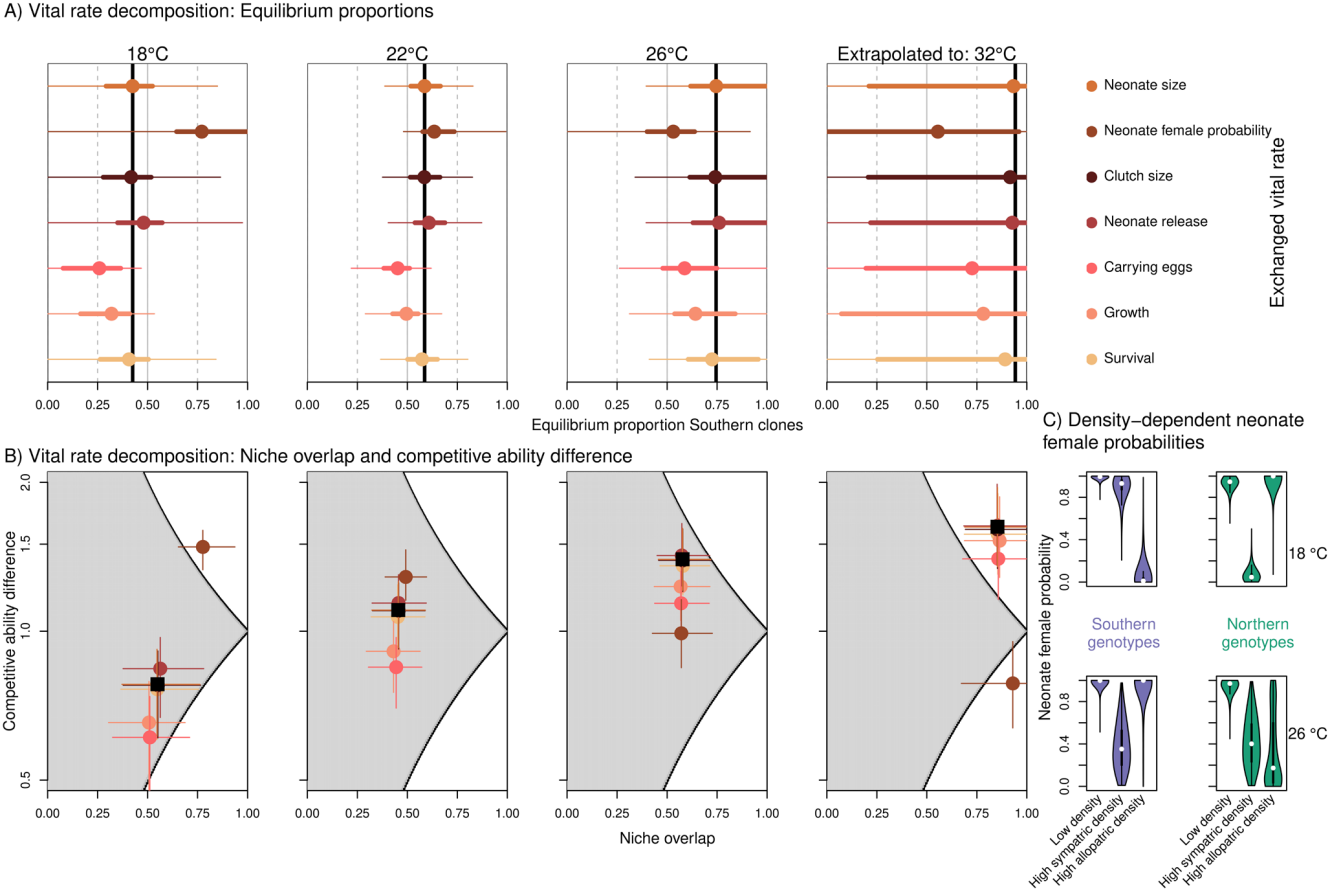
Decomposition analysis reveals neonate female probabilities as an important driver in the temperature‐dependent coexistence outcomes. (A) Decomposition analysis showing the contribution of each vital rate to the predicted competition outcomes, at different temperatures. In each panel, vertical black lines show the median predicted equilibrium proportion of the Southern genotypes, at the relevant temperature (matching red dots in Figure 2E). Coloured dots show the median equilibrium proportion when we exchanged one of the vital rates. To this end, we assigned the Southern genotypes the vital rate function of the Northern genotypes, and vice versa. Whenever this increases the proportion of Southern genotypes, it indicates a Northern demographic advantage for the specific vital rate. On the other hand, whenever it decreases the proportion of Southern genotypes, it indicates a Southern demographic advantage. Thick and thin lines show 0.25–0.75 and 0.05–0.95 quantiles respectively, based on 1000 draws from the posterior distributions in each vital rate. (B) Vital rate contributions to predicted niche overlap and competitive ability difference. Black squares indicate the median niche overlap and competitive ability difference across 1000 posterior draws. Coloured dots show the median competition outcomes when we exchanged one of the vital rates (colours match A). Horizontal and vertical lines indicate 0.25–0.75 quantiles, based on 1000 posterior draws. (C) Predicted neonate female probabilities based on the fitted vital rate model, for different combinations of sympatric and allopatric density, and shown for two temperatures. Low densities correspond to 1 individual; high densities are based on the 90th percentile of the observed population densities, corresponding to 459 individuals. Violin plots show distributions based on 10,000 draws from each posterior predictive distribution.

Quantifying the contributions of each vital rate in driving these temperature‐dependent coexistence outcomes reveals that the eggs‐carrying probability and somatic growth confer consistent advantages to the Southern genotypes: At all tested temperatures, the equilibrium proportions of Southern genotypes would decline if their vital rates were replaced with those of the (average) Northern genotypes (Figure 4A). In contrast, no vital rate provided a consistent advantage to Northern genotypes. A notable example is the impact of female neonate production on competitive outcomes, which interacts with temperature: at low temperatures, Southern genotypes benefit (i.e., increase in proportion) from adopting the Northern female neonate production rates, while the reverse emerges at higher temperatures. Across temperatures, vital rate contributions to competition outcomes mainly act through competitive ability differences (equalisation), and not niche overlap (stabilisation) (Figure 4B).

These results stem from the interaction between latitude, temperature and (allopatric and sympatric) density. At low densities, female neonate probability is ~1 across temperatures and latitudes (Figure 4C). At low temperatures, increasing Northern genotype densities disproportionately promote male neonate production of Southern genotypes. In contrast, at high temperatures, Southern genotypes show minimal change in neonate sex ratio with increasing allopatric density, while Northern genotypes respond strongly. On the population level, these temperature‐dependent density responses drive the temperature‐dependent coexistence outcomes.

## 4 Discussion

Rising temperatures drive genetic shifts in local populations (Parmesan 2006), that may be further altered by competition with migrating conspecifics better suited to the changing environment (Alexander et al. 2015). The impact of interactions with sympatric and allopatric conspecifics depends on how competition (or facilitation) affects individuals across their life‐cycle and how these effects integrate at the population level, features that are rarely studied (Lyu and Alexander 2023). We make use of an Integral Projection Modelling framework to do so, based on the theoretical foundations of modern coexistence theory (Box 1; Figure 1). This IPM framing allows for a straightforward interpretation analogous to that of a Lotka‐Volterra competition model, but with deeper mechanistic and biological insights into the underlying individual vital rates that shape competition outcomes.

### 4.1 Explaining Coexistence Among Genotypes

Most vital rates were negatively associated with (sym‐ and allopatric) population densities (Bruijning, tenBerge, and Jongejans 2018) (Appendix 2.3). Specifically, higher densities decreased growth and reproduction, as expected from a decrease in per capita food availability (Lampert 1978). At the same time, increased densities led to increased *Daphnia* neonate size, in line with positive associations between food stress and neonate size, which is an adaptive plastic response that leads to a higher starvation resistance (Guisande 1993).

The long‐term co‐occurrence of multiple asexual genotypes, such as zooplanktonic *Daphnia*, has been coined ‘another plankton paradox’ (Hebert and Crease 1980), referencing the plankton paradox (Hutchinson 1961). This co‐occurrence implies either slow exclusion or stable coexistence. MCT predicts stable coexistence when individuals experience stronger competition from their own genotype than their impact on other genotypes. By integrating vital rate‐specific density effects across life‐cycles, we detected stronger sympatric than allopatric competition, predicting—on average—stable coexistence of Northern and Southern genotypes across all tested treatments (18°C–26°C) (Figures 2 and 3). Estimating interactions in communities with many combinations of competing pairs is a well‐known experimental and statistical challenge (Brown and Stouffer 2025; Ovaskainen et al. 2017). We therefore made the simplifying assumption that competition strength differs among latitudes, but is the same for genotypes within a latitude. This is based on the expectation that ecological divergence leads to more similar ecological traits within than among latitudes. Therefore, individuals from the same latitude are expected to be more similar in terms of their responses to competition, compared to individuals from different latitudes. As a consequence, our models overlook any within‐latitudinal stabilisation. While this is not likely to have an effect on our cross‐latitudinal results, our models do not provide any insights into within‐latitude coexistence outcomes.

Various biological processes can contribute to stronger sympatric than allopatric competition. Different asexual genotypes may occupy different niches (Vrijenhoek 1998). The observation that *Daphnia* genotypes vary in their responses to stoichiometric food conditions (Weider et al. 2005), and utilise sediments to different extents (Arbore et al. 2016), is suggestive of differential resource utilisation. Further, individuals may directly interfere. In *Daphnia*, both physical interactions (Ban et al. 2008, 2009) and the production of chemicals or metabolites (Helgen 1987; Lürling et al. 2003; Matveev 1993) can lead to interference and resulting density dependence. We are not aware of any studies comparing the strength of such crowding factors within and among genotypes (but see Hobaek and Larsson (1990) for a study on crowding effects among different *Daphnia* species). Finally, while not playing a role in this study, specialised pathogens are major drivers of frequency‐dependent selection, affecting dominant genotypes disproportionally so that rare genotypes have a fitness advantage (Wolinska and Spaak 2009), also fostering stable coexistence.

### 4.2 Temperature‐Dependent Coexistence and Thermal Adaptation

Even though applying MCT to our IPMs led, on average, to predictions of stable coexistence across temperatures, temperature certainly altered the competition outcomes (Appendix 4, Figures 2 and 3). The mechanisms by which temperature mediates individual interactions influence the dynamics at the population level (Amarasekare and Coutinho 2014; Johnson et al. 2016; Mallard et al. 2020). For various vital rates, we found that increasing temperatures intensified sym‐ and allopatric competition (i.e., significant interactions between temperature and sym‐ and allo‐patric densities). This is likely caused by higher individual metabolic rates, but constant food availability across temperatures. At the population level, this led to lower carrying capacities (Bernhardt et al. 2018), both predicted with our IPM and observed in our data (Figure 2; Appendix 1).

Temperature‐mediated competition differently impacted Northern and Southern genotypes. Results suggest the presence of local adaptation, where genotypes from warmer climates perform better at high temperatures compared to genotypes from colder climates (Geerts et al. 2015; Yampolsky et al. 2014): Southern genotypes became more dominant at higher temperatures, supported by observed shifts in genotype frequencies (Figures 2 and 3).

Observed local thermal adaptation and predicted shift in competitive outcomes became apparent only through our density‐dependent vital rates integration. No single vital rate stood out showing a clear latitude × temperature interaction. Moreover, even predicted intrinsic growth rates of a single individual, based on a vital rate integration, did not reveal the subtle two‐directional thermal adaptation: Southern intrinsic growth rates exceeded Northern's across temperatures (Appendix 5). Thus, life table experiments or single genotype population experiments would not have revealed the temperature‐dependent coexistence outcomes, highlighting the importance of competition experiments. The challenge in predicting community composition based on monoculture species performance is well‐known (Fu et al. 2020; Tilman et al. 2001). We show that predicting evolutionary shifts faces the same challenge, and that density‐dependent demographic modelling can help gain insight into coexistence mechanisms.

### 4.3 Investment in Asexual and Sexual Reproduction

Disentangling the vital rates causing temperature‐mediated competition outcomes highlighted neonate female probability as an important vital rate, mainly through impacts on competitive ability differences (Figure 4). The production of *Daphnia* males, as a first step towards sexual reproduction, is increased when females experience less favourable environments, responding to cues like temperature, photoperiod and population density (Korpelainen 1986; Wood and Banta 1933). In line with this, the observed female: male neonate ratio was essentially 1:0 at low densities, becoming increasingly male‐biased at higher densities (Appendix 2.5). Interestingly, at low temperatures, Northern genotypes started producing males at lower sympatric densities than the Southern genotypes, with this effect being reversed at high temperatures (Figure 4C). Investment in sexual production through the production of males and of sexual dormant eggs (ephippia) has been shown to be aligned (Roulin et al. 2015). Indeed, modelled neonate male production matches observed ephippia counts (Appendix 6): Northern genotypes produced the most ephippia at low temperatures, but almost none at high temperatures, while Southern genotypes show the opposite.

Latitude × temperature × density interactions for neonate sex ratios led to a demographic benefit to the Northern genotypes at low temperatures, while this was reversed at high temperatures (Figure 4). Because of the strong density dependence in sex ratios, life table experiments monitoring single individuals would completely overlook this vital rate. We note that uncertainty in all vital rates combined led to substantial variation in the effects of exchanging Northern and Southern neonate sex ratios, reflected by 95% credible intervals overlapping with the median predicted equilibrium proportions (Figure 4A). This uncertainty is especially apparent for predictions at 32°C, where we extrapolated beyond the tested temperature range. In addition, model selection was not able to confidently identify that the inclusion of the latitude × temperature × density interactions brought additional predictive capacity (Appendix 2.2). This warrants future studies to unravel density‐ and temperature‐dependent genotypic variation in neonate sex ratio in an experimental set‐up that controls sym‐ and allopatric densities.

In our experiments and IPM, only the production of (parthenogenetic) female neonates contributed to population growth. Our study thus excluded any long‐term fitness advantage of sexual reproduction, instead focusing on the dynamics within one growing season; sexual investment does not contribute to population growth within a growing season. As a consequence, a genotype investing early in sexual reproduction will reach lower abundances at the end of the season compared to a genotype that invests in sexual reproduction later (Fossen et al. 2021; Gerber, Booksmythe, and Kokko 2018). However, as populations approach their carrying capacities, individuals will experience stronger density dependence. Due to diminishing returns of asexual reproduction, relative demographic costs of sexual reproduction will decrease (Gerber, Kokko, et al. 2018), shifting selection towards sexual reproduction. In habitats that become temporarily unfavourable, for example, due to low winter temperatures or drying up of the water body, *Daphnia* cannot survive year‐round. Under those conditions, investing in sexual reproduction eventually, through the production of fertilised dormant eggs, is essential to contribute to the gene pool in the next season. If season duration is unpredictable, spreading the timing of sexual production may optimise long‐term fitness, representing a bet hedging strategy (Gerber, Booksmythe, and Kokko 2018). Also, by producing dormant eggs early in the season, the less competitive genotype may even prevent exclusion in the long run (Maruoka et al. 2024). These different dimensions of long‐term fitness result in different strategies for the investment in sexual reproduction, varying across and within populations from different habitats and regions. Incorporating such long‐term fitness would require experiments mimicking multiple bouts of parthenogenetic and sexual reproduction, which is beyond the scope of the current study.

### 4.4 Conclusions

Understanding population persistence under climate change is challenging, especially as impacts of migrating competitors may exceed those of warming alone. While modern coexistence theory offers a framework for population‐level outcomes, it often overlooks individual life histories. Moreover, empirical studies applying MCT are mostly limited to primary producers. Here, we link individual‐ and population‐level processes to gain more mechanistic insights into coexistence of primary consumers originating from different climatic zones. Our models predict that the neonate female probability may play a crucial role in determining competition outcomes by shifting competitive ability differences between Northern and Southern clones in a temperature‐dependent way. Future experiments designed to specifically test how Northern and Southern clones differ in their reproductive strategies will elicit the role of this specific and often‐ignored vital rate to competition outcomes between clones from different latitudes. While we focused on the dynamics of pairs of competing genotypes, we hope that future studies can extend our approach to diverse communities with more complex dynamics, benefiting from theoretical advances (Spaak et al. 2021, 2023). Altogether, this study underscores the importance of integrating individual life histories with population‐level processes to understand evolutionary and ecological responses to environmental change.

## Author Contributions

M.B., L.D.M., M.D.V. and E.J. designed the modelling framework. M.B. performed all analyses. M.B., E.I.F.F., J.A.M.R., S.E., L.D.M. and E.J. designed the experiments. M.B., E.I.F.F. and H.V. collected data, with help from L.G. and K.I.B. M.B. wrote the first draft of the manuscript, and all authors contributed substantially to revisions.

## Peer Review

The peer review history for this article is available at https://www.webofscience.com/api/gateway/wos/peer‐review/10.1111/ele.70214.

## Supporting information


**Data S1:** ele70214‐sup‐0001‐Supinfo.pdf.

## Data Availability

Data and code are available at Zenodo (https://doi.org/10.5281/zenodo.17073497).
