# Supplementary material for: Linking Individual Performance to Density‐Dependent Population Dynamics to Understand Temperature‐Mediated Genotype Coexistence
Source: Ecol Lett. 2025 Sep 15;28(9):e70214. doi: 10.1111/ele.70214 (PMC12436995; doi:10.1111/ele.70214)

Linking individual performance to density-dependent population dynamics to understand temperature-mediated genotype coexistence

Supplementary Information

Marjolein Bruijning   Luc De Meester   Marco D. Visser   Erlend I.F. Fossen
Helene Vanvelk   Joost A.M. Raeymaekers   Lynn Govaert   Kristien I. Brans
Sigurd Einum   Eelke Jongejans

Contents

1 Population densities **2**

2 Vital rates **3**
2.1 Tested models 3
2.2 Model selection 3
2.3 Estimated coefficients 5
2.4 Posterior distributions 6
2.5 Vital rate visualization 13
2.6 Model fit 17

3 Variation among genotypes on competition outcomes **20**

4 Genotype frequency model **22**

5 Population growth rates at low density **23**

6 Produced ephippia per population **24**

1 Population densities

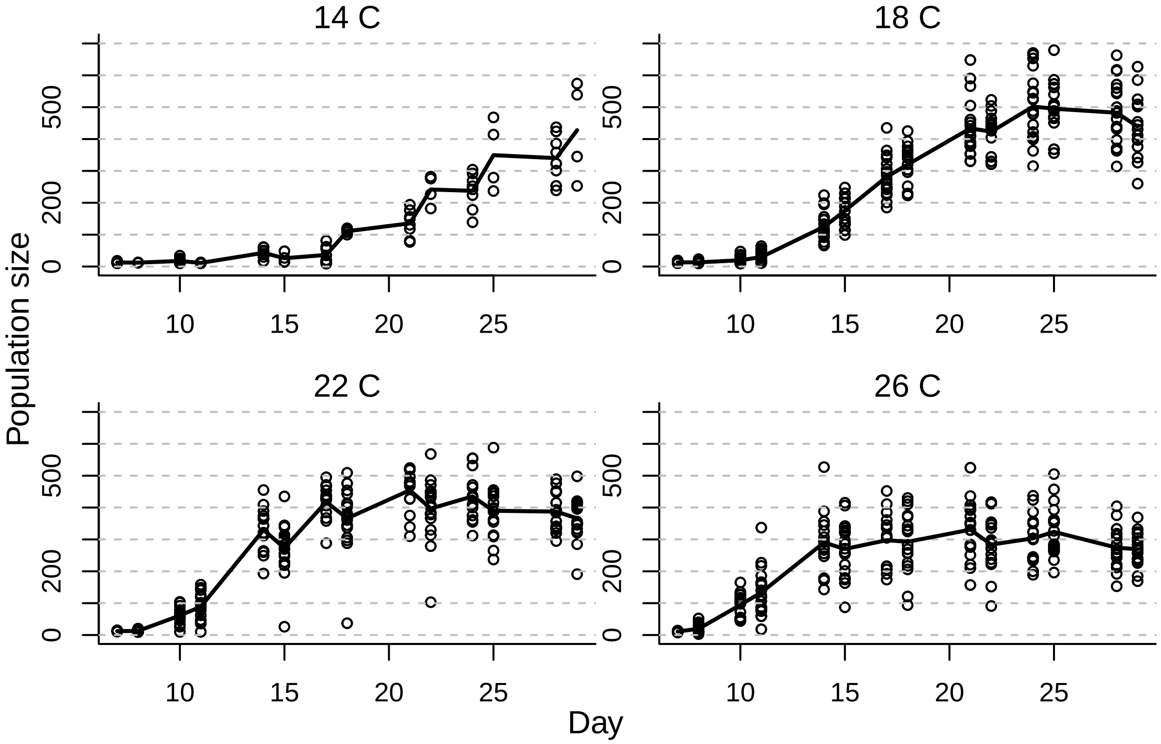

Figure 1: Observed populations numbers across all populations, plotted for each temperature. Dots show observations, lines show averages.

2 Vital rates

2.1 Tested models

Table 1: Overview of the fixed part of the fitted generalized linear models models, fitted per vital rate. Note that in the case of survival and somatic growth, models included an effect of sex, resulting in an additional parameter. Appropriate link functions were used, depending on the vital rate, as described in the manuscript.

Description	Model
Additive effects of body size (z), squared body size (z^2), temperature (T), latitude (L) and sym- and allopatric density (S and A, respectively) (7 parameters).	$y = \beta_0 + \beta_z z + \beta_{z^2} z^2 + \beta_T T + \beta_L L + \beta_S S + \beta_A A$
Starting from 1), this model includes interactions between temperature and sym- and allopatric density density (9 parameters).	$y = \beta_0 + \beta_z z + \beta_{z^2} z^2 + \beta_T T + \beta_L L + \beta_S S + \beta_A A + \beta_{TS} TS + \beta_{TA} TA$
Starting from 1), this model includes interactions between latitude and sym- and allopatric density density (9 parameters).	$y = \beta_0 + \beta_z z + \beta_{z^2} z^2 + \beta_T T + \beta_L L + \beta_S S + \beta_A A + \beta_{LS} LS + \beta_{LA} LA$
Starting from 3), this model includes interactions between temperature and sym- and allopatric density density (11 parameters).	$y = \beta_0 + \beta_z z + \beta_{z^2} z^2 + \beta_T T + \beta_L L + \beta_S S + \beta_A A + \beta_{LS} LS + \beta_{LA} LA + \beta_{TS} TS + \beta_{TA} TA$
Starting from 1), this model includes three-way interaction between latitude, temperature, and both sym- and allopatric density (14 parameters).	$y = \beta_0 + \beta_z z + \beta_{z^2} z^2 + \beta_T T + \beta_L L + \beta_S S + \beta_A A + \beta_{LS} LS + \beta_{LA} LA + \beta_{TS} TS + \beta_{TA} TA + \beta_{TLS} TLS + \beta_{TLA} TLA$

2.2 Model selection

In the tables below we compare the predictive accuracy of the different fitted models, per vital rate, expressed as the difference in ELPD (expected log-predictive density), and its standard error. The model numbers correspond to numbers shown in Table 1 (Appendix 2.1). For each vital rate, we selected the model with the highest predictive accuracy (top row in each table).

Table 2: Model comparison for: Survival

	elpd_diff	se_diff
1	0.000000	0.0000000
3	-0.019597	0.9347437
2	-1.881060	2.2425715
4	-2.238128	2.1964187
5	-4.865533	3.2630074

Table 3: Model comparison for: Growth

	elpd_diff	se_diff
1	0.000000	0.0000000
2	-1.106409	1.5141022
3	-1.685777	0.7004194
5	-2.429691	3.2790553
4	-2.902948	1.5865150

Table 4: Model comparison for: Carrying eggs

	elpd_diff	se_diff
4	0.0000000	0.000000
2	-0.8618361	2.352963
5	-2.0900348	1.697025
3	-2.7838303	2.894402
1	-3.5955177	3.862061

Table 5: Model comparison for: Neonate release

	elpd_diff	se_diff
3	0.000000	0.000000
4	-2.354344	1.425007
1	-2.539183	2.062380
2	-4.872087	2.544582
5	-5.093599	2.669706

Table 6: Model comparison for: Clutch size

	elpd_diff	se_diff
2	0.0000000	0.000000
5	-0.2026644	11.710654
4	-2.6256895	4.805086
1	-53.6572082	24.081938

	elpd_diff	se_diff
3	-58.4416136	23.650740

Table 7: Model comparison for: Offspring female probability

	elpd_diff	se_diff
5	0.0000000	0.000000
3	-0.5762865	5.158478
4	-2.9479424	5.293031
1	-4.5546359	6.124888
2	-6.8451958	6.043501

Table 8: Model comparison for: Neonate size

	elpd_diff	se_diff
2	0.0000000	0.0000000
1	-0.5759429	2.4393647
4	-1.8872657	0.3641084
3	-2.3921301	2.4812879
5	-5.4698906	0.7677832

2.3 Estimated coefficients

Table 9: The median of the posterior distribution of each parameter, and for each vital rate. Parameters in bold indicate that the 95% credible intervals did not include 0, see Section 2.4 for full posterior distributions. Missing values indicate that the vital rate model did not include that specific coefficient based on the model selection procedure, see also Section 2.2. Parameter subscripts indicate the covariates (and their interactions) for which the parameters are being estimated: body size (z), squared body size (z^2), temperature (T), latitude (L) and sym- and allopatric density (S and A, respectively).

	β_0	β_z	β_{z^2}	β_T	β_L	β_M	β_S	β_A	β_{TS}	β_{LS}	β_{TA}	β_{LA}	β_{LT}	β_{TLS}	β_{TLA}
Survival	**5.5011**	-0.3131	**-0.7716**	-0.2662	-0.1891	0.3229	-0.0010	0.0081	NA	NA	NA	NA	NA	NA	NA
Growth	**0.1265**	**-0.0534**	**0.0049**	-0.0025	**-0.0187**	**-0.0619**	**-0.0001**	**-0.0001**	NA	NA	NA	NA	NA	NA	NA
Carrying eggs	-0.5687	**4.9640**	**-1.6274**	0.2665	0.6996	NA	**-0.0055**	-0.0009	**-0.0018**	**-0.0031**	-0.0032	-0.0032	NA	NA	NA
Egg development	**2.1245**	0.7195	-0.2348	**0.6637**	-0.3766	NA	-0.0023	0.0020	NA	**0.0059**	NA	**0.3188**	NA	NA	NA
Clutch size	**1.7423**	**1.4737**	-0.1390	**0.1247**	-0.2441	NA	**-0.0037**	**-0.0045**	**-0.0013**	NA	**-0.0036**	NA	NA	NA	NA
Offspring sex	**6.7620**	-1.7408	0.8140	0.6626	-2.6710	NA	**-0.0098**	-0.0113	-0.0050	-0.0015	0.0127	**0.0160**	-0.3430	0.0072	**-0.0256**
Neonate size	**-1.7020**	**0.3862**	**-0.1716**	**0.1263**	-0.0878	NA	**0.0003**	**0.0006**	0.0001	NA	0.0006	NA	NA	NA	NA

2.4 Posterior distributions

In the figures below, we show posterior distributions for each parameter and each vital rate. Red lines indicate median values, corresponding to the values shown in Table 9 (Appendix 2.3).

2.4.1 Survival

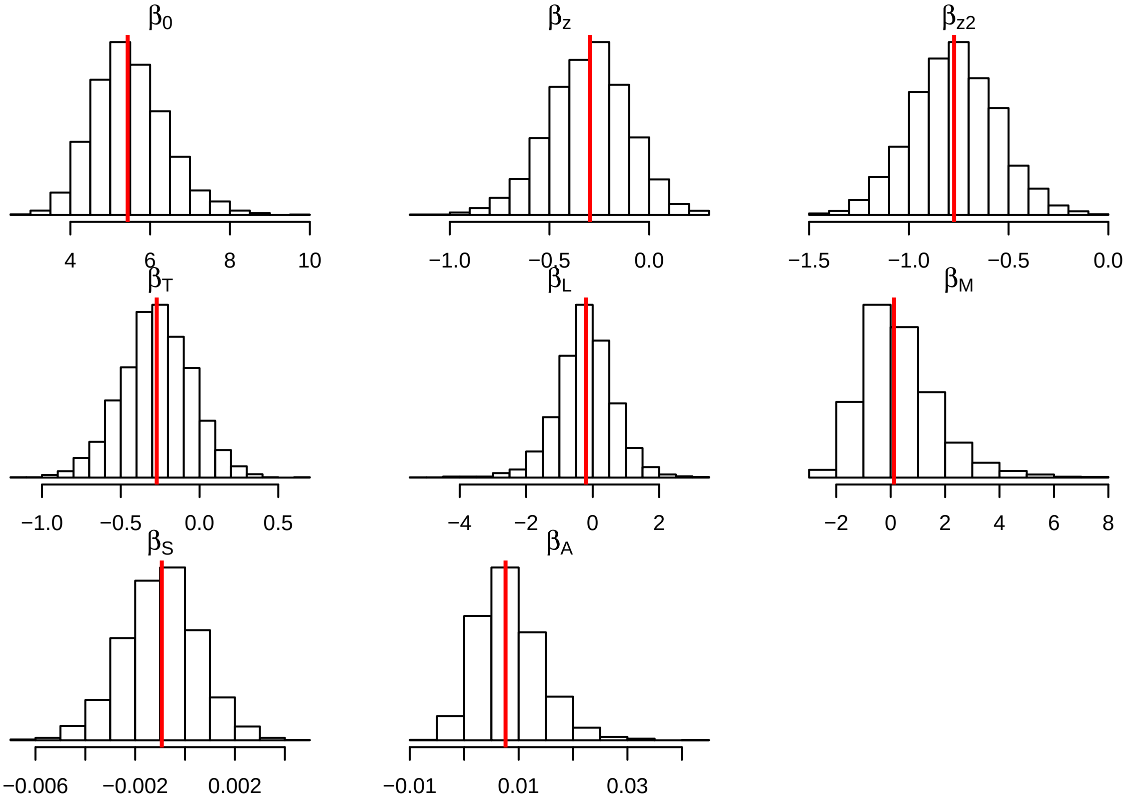

2.4.2 Growth

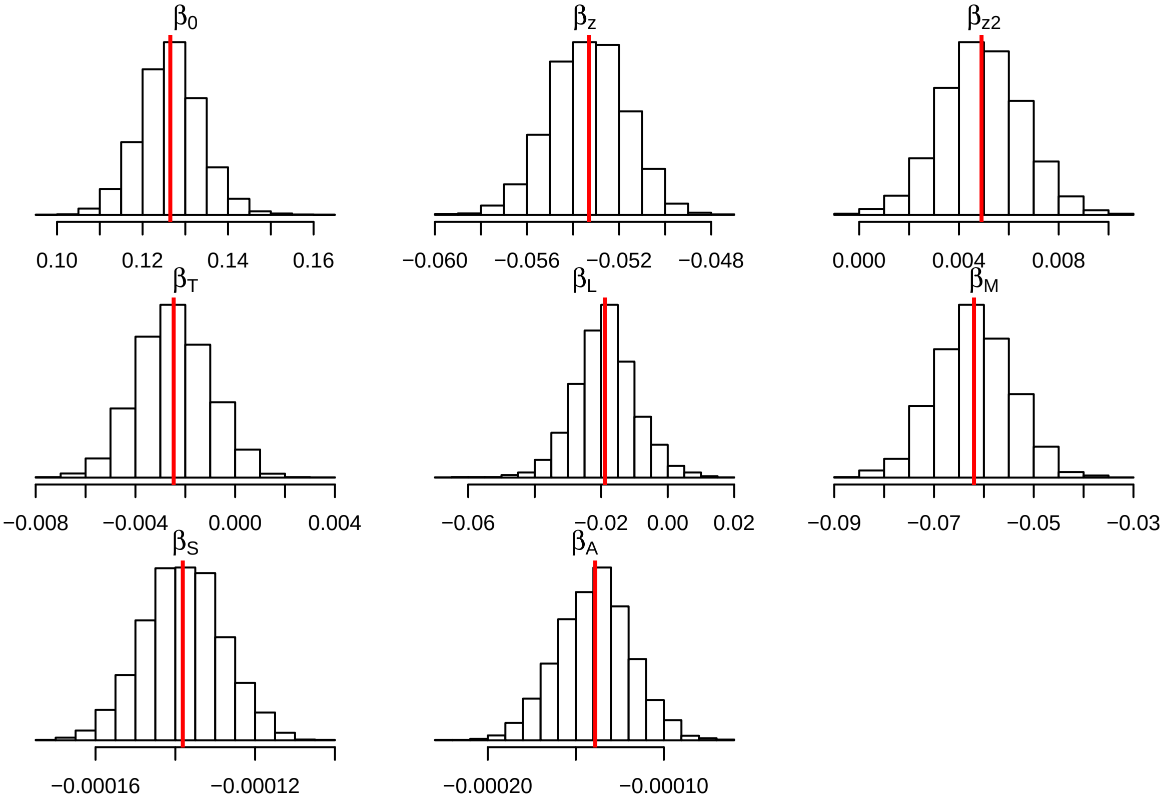

β0
0.10 0.12 0.14 0.16
βz
−0.060 −0.056 −0.052 −0.048
βz2
0.000 0.004 0.008
βT
−0.008 −0.004 0.000 0.004
βL
−0.06 −0.02 0.00 0.02
βM
−0.09 −0.07 −0.05 −0.03
βS
−0.00016 −0.00012
βA
−0.00020 −0.00010

2.4.3 The probability of carrying eggs

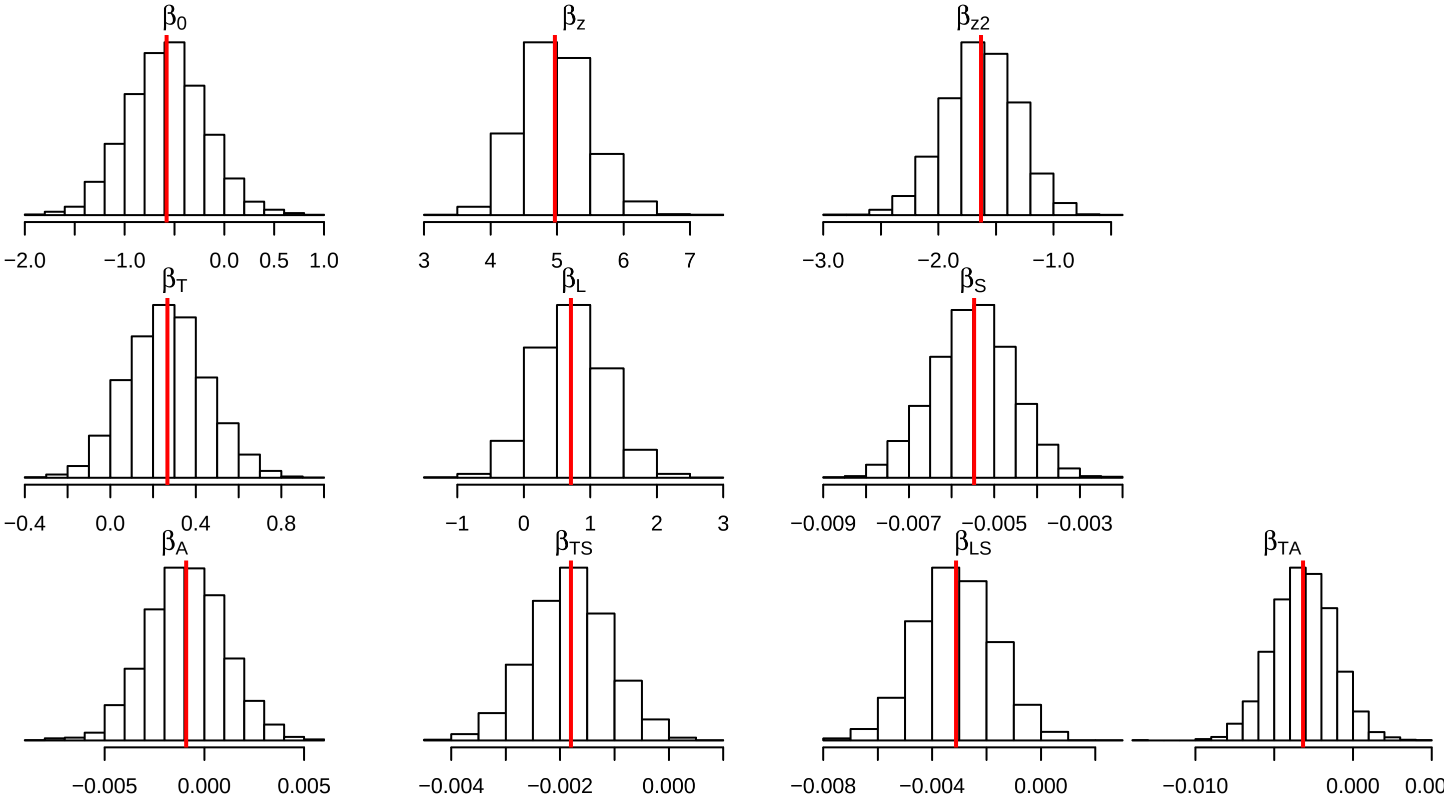

2.4.4 The probability of producing offspring

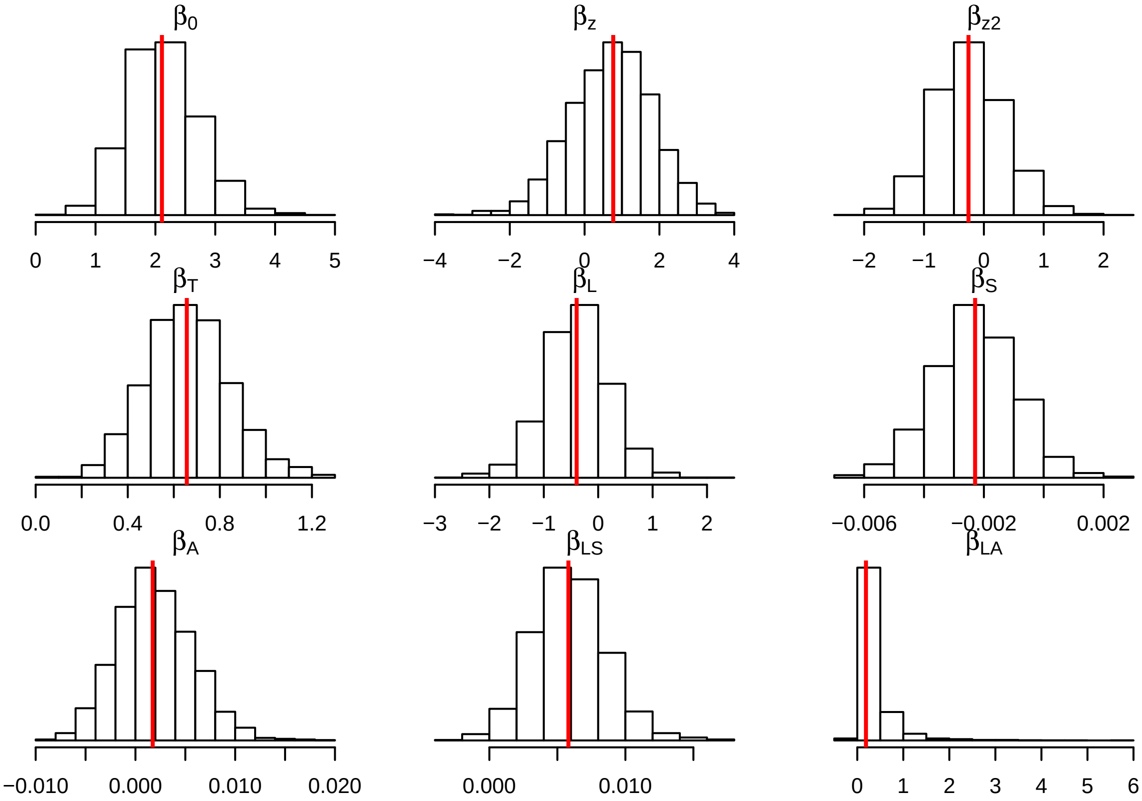

2.4.5 Clutch size

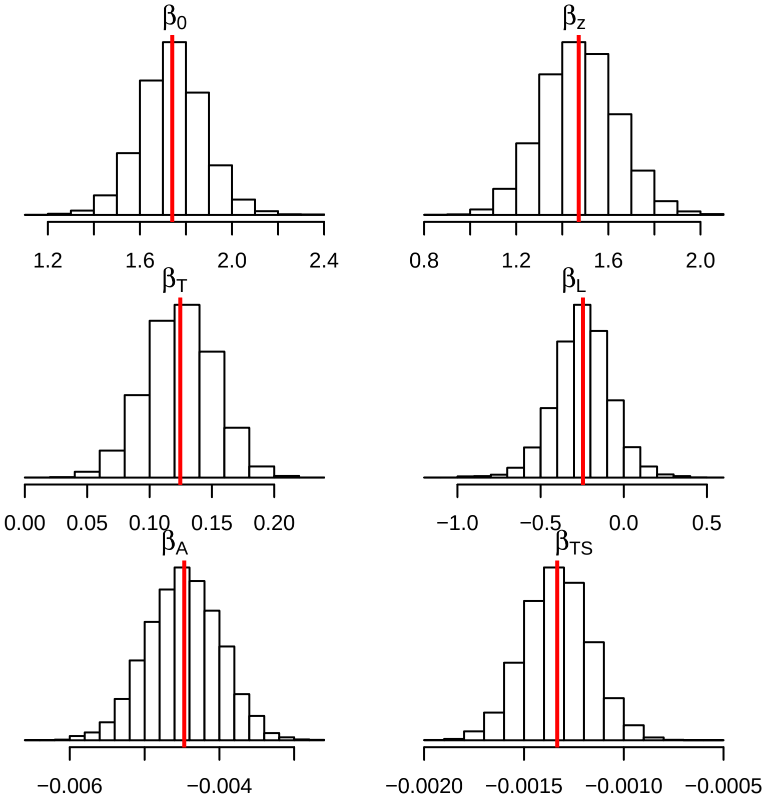

2.4.6 Neonate female probability

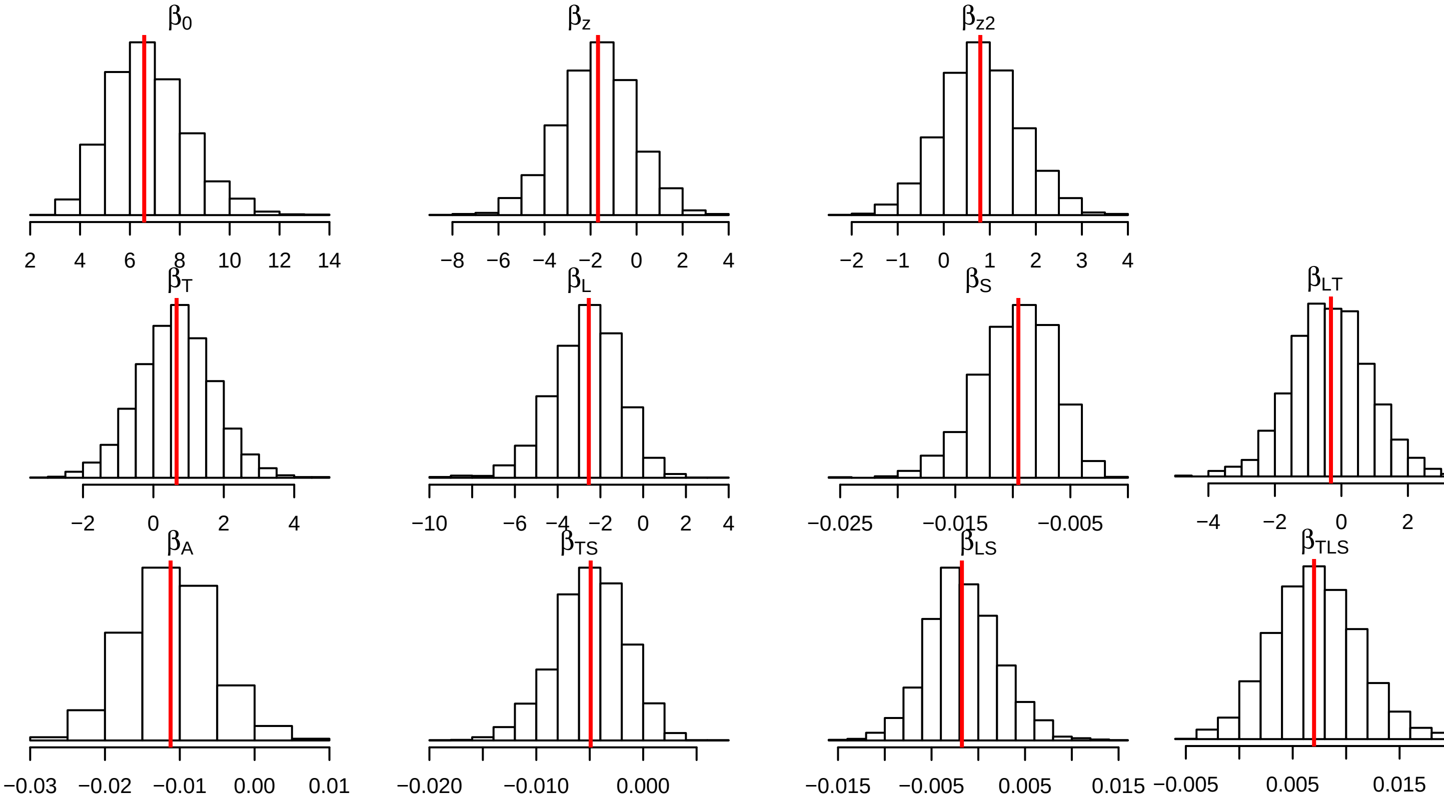

2.4.7 Offspring body size

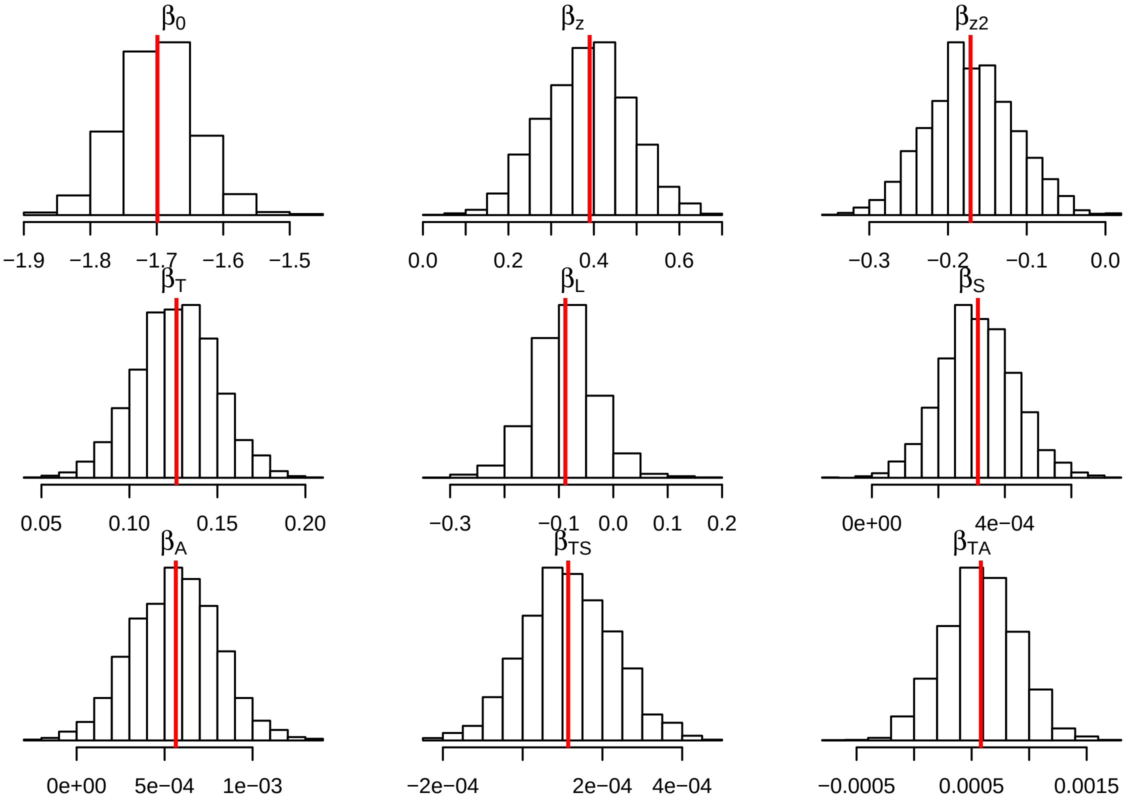

2.5 Vital rate visualization

Below we visualize each vital rate, setting standardized body size to 1 SD above the mean (corresponding to 2.9 mm), temperature to 18 ^{o}C and 26 ^{o}C, shown in blue and red colors, respectively. We visualize the mean predictions as a function of either sympatric (left column) or allopatric densities (right column), setting the other density at 0. Solid lines show mean predictions for the Northern genotypes; dotted lines show mean predictions for the Southern genotypes. Dots show partial residuals (accounting for all other variables; circles: Northern genotypes; triangles: Southern genotypes), averaging per density class. Dots are scaled to the number of datapoints (log-transformed). In the left column, we provide the estimated 95% credible intervals of the estimated additive effects of temperature T, latitude L (estimating the effect of Northern latitude compared to Southern latitude), sympatric density S and allopatric density A. In bold indicates whenever 95% credible intervals excluded zero.

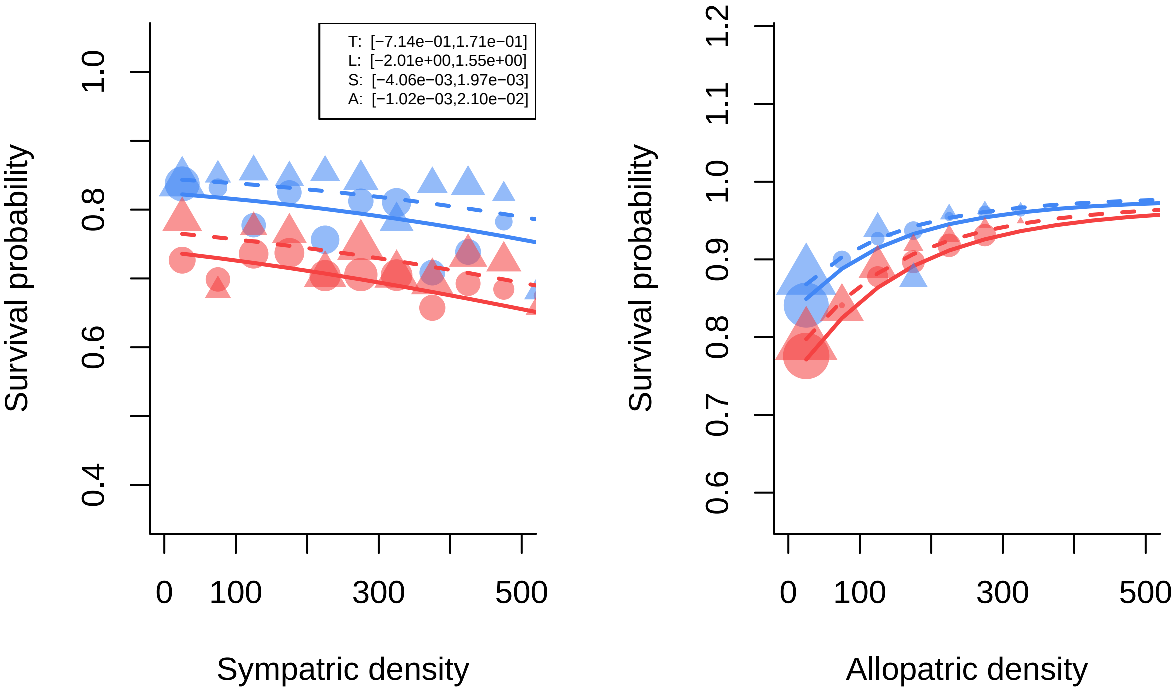

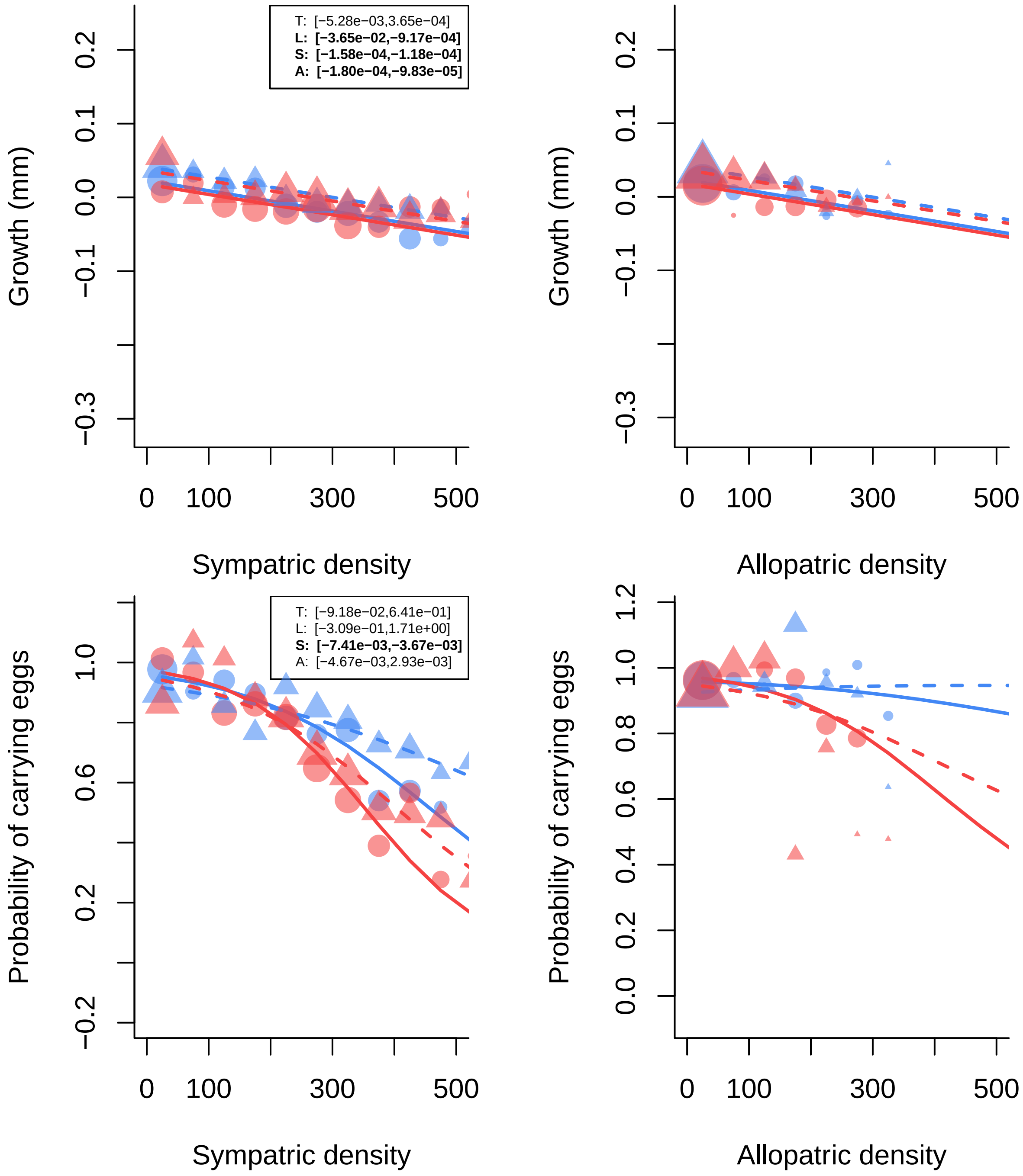
T: [−5.28e−03,3.65e−04]
L: [−3.65e−02,−9.17e−04]
S: [−1.58e−04,−1.18e−04]
A: [−1.80e−04,−9.83e−05]
Growth (mm)
Sympatric density
Allopatric density
T: [−9.18e−02,6.41e−01]
L: [−3.09e−01,1.71e+00]
S: [−7.41e−03,−3.67e−03]
A: [−4.67e−03,2.93e−03]
Probability of carrying eggs

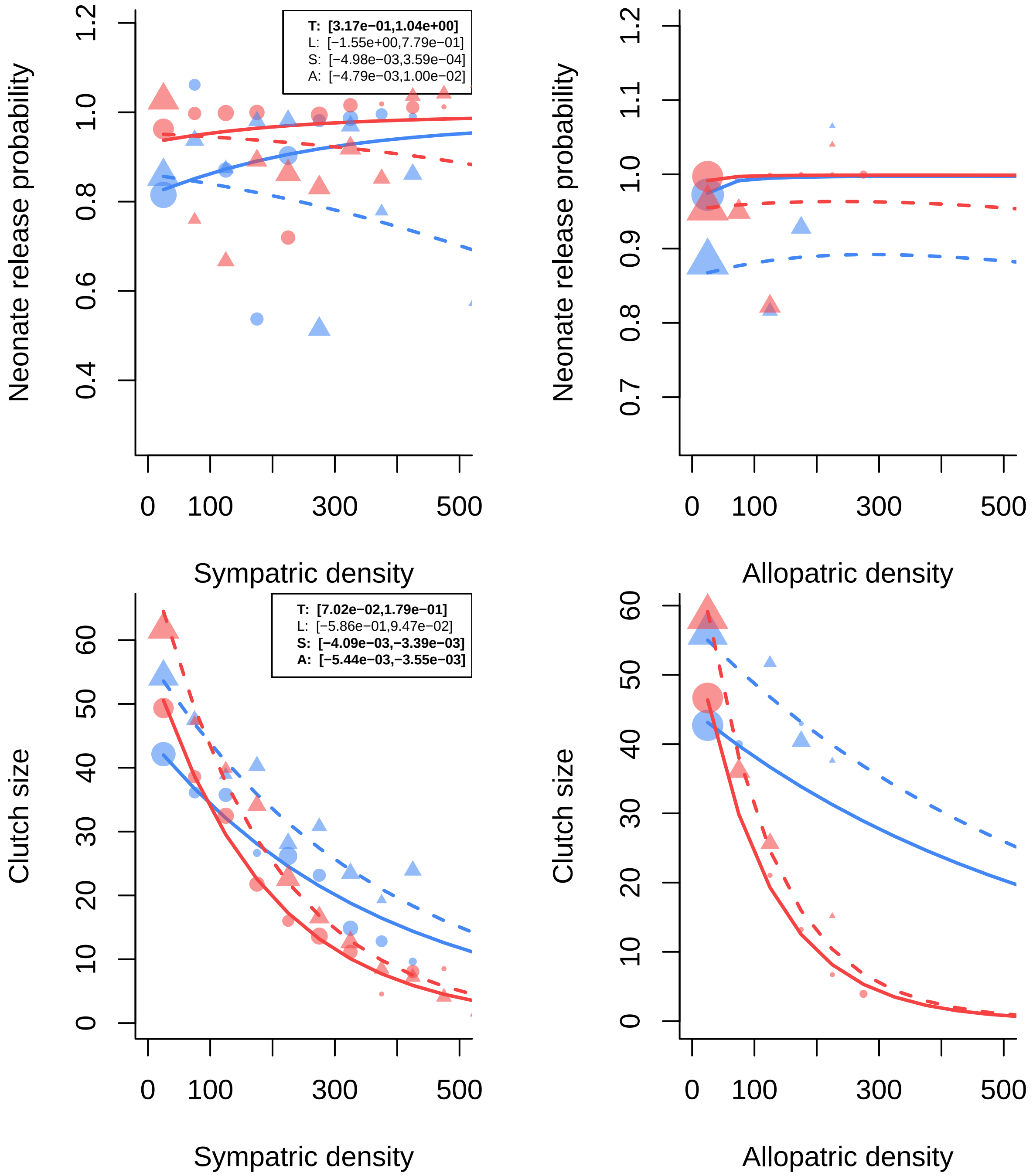

Neonate release probability
T: [3.17e−01,1.04e+00]
L: [−1.55e+00,7.79e−01]
S: [−4.98e−03,3.59e−04]
A: [−4.79e−03,1.00e−02]
Sympatric density
Allopatric density
Clutch size
T: [7.02e−02,1.79e−01]
L: [−5.86e−01,9.47e−02]
S: [−4.09e−03,−3.39e−03]
A: [−5.44e−03,−3.55e−03]

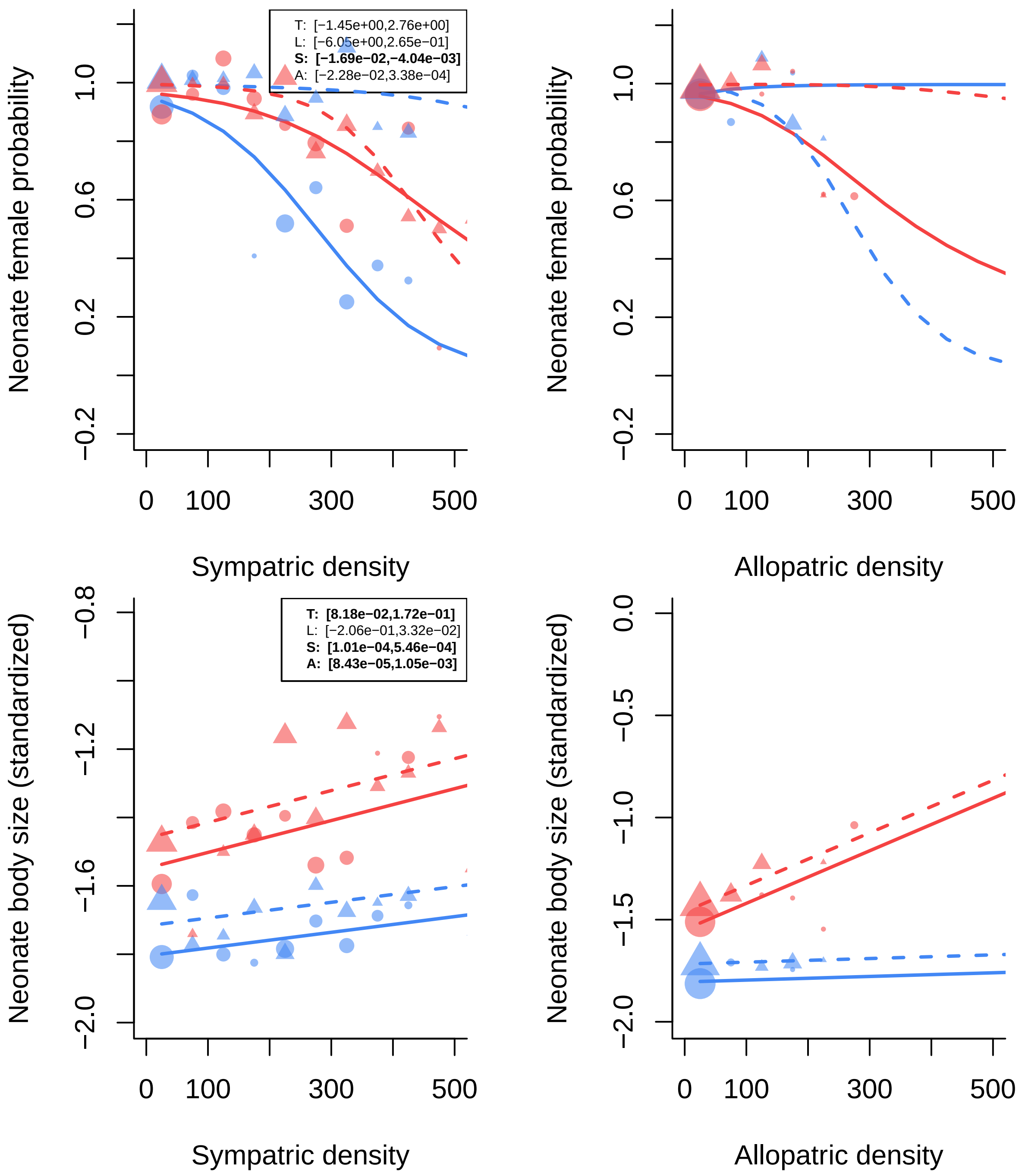
Neonate female probability
Sympatric density
Allopatric density
Neonate body size (standardized)
T: [−1.45e+00,2.76e+00]
L: [−6.05e+00,2.65e−01]
S: [−1.69e−02,−4.04e−03]
A: [−2.28e−02,3.38e−04]
T: [8.18e−02,1.72e−01]
L: [−2.06e−01,3.32e−02]
S: [1.01e−04,5.46e−04]
A: [8.43e−05,1.05e−03]

2.6 Model fit

To confirm that the linear models that we used capture our data, we here show the following figures, per vital rate: 1) We show all raw data points, structured by body size and colored by temperature (dark blue: 14 ^oC; light blue: 18 ^oC; orange: 22 ^oC; red: 26 ^oC); 2) We show the residuals, again structured by body size; 3) We plot predicted against observed values. In 2) and 3), we add smoothened curves (in black), showing that, despite considerable variation in our vital rates (as expected from demographic data that are inherently noisy), residuals are independent of body size (1) and that predictions and observations show a linear relationship (2).

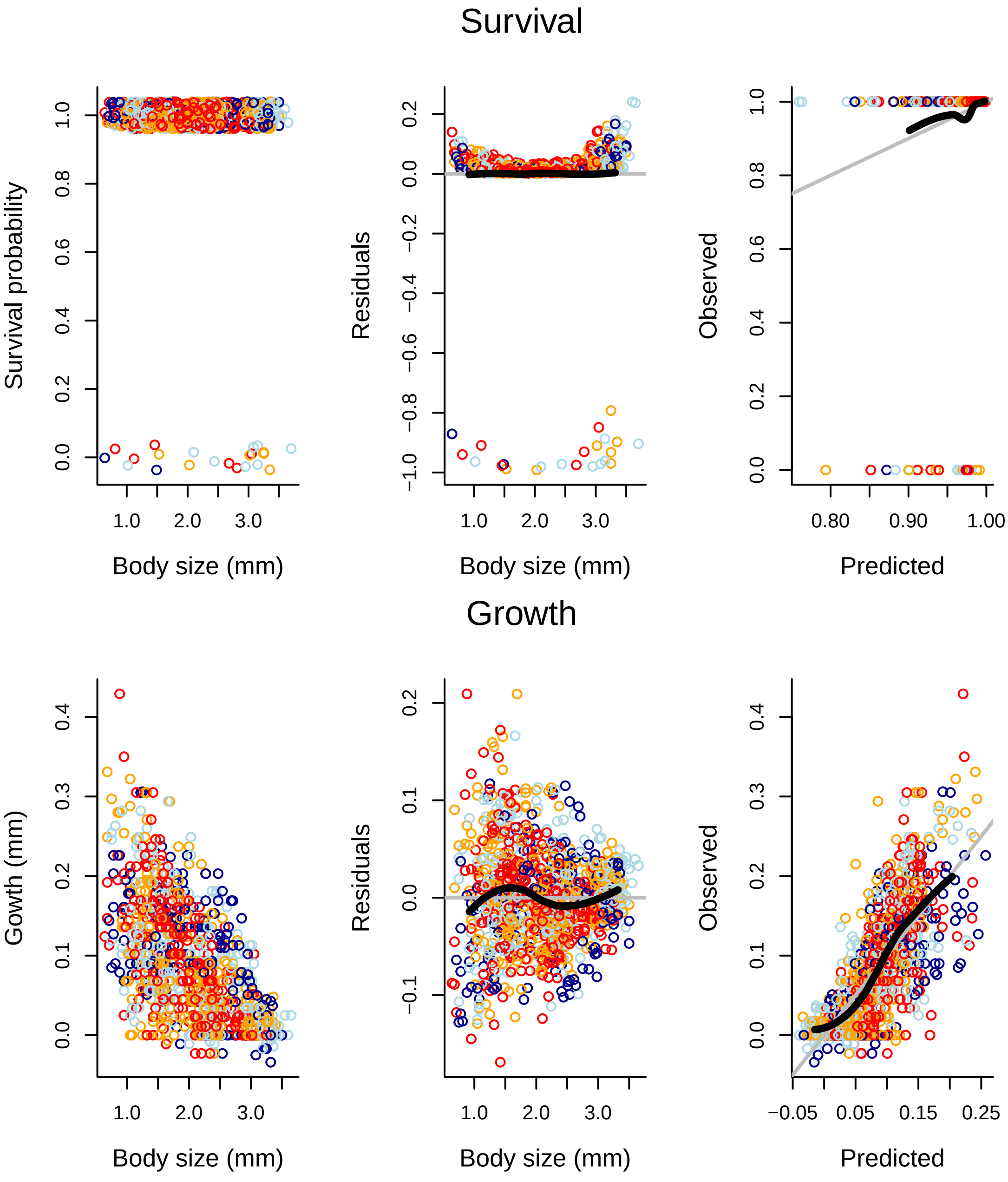

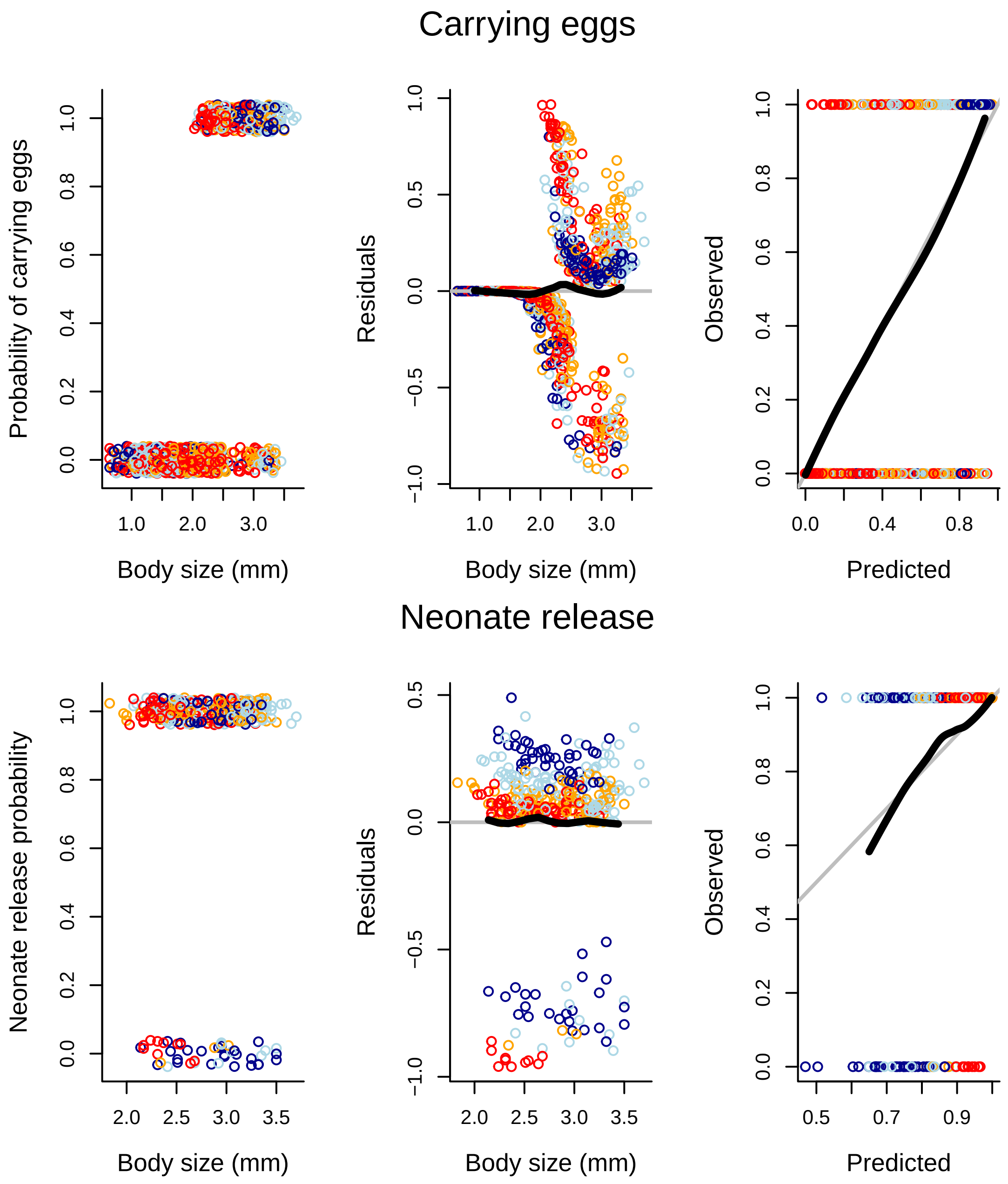
Carrying eggs
Probability of carrying eggs
Body size (mm)
Residuals
Body size (mm)
Observed
Predicted
Neonate release
Neonate release probability
Body size (mm)
Residuals
Body size (mm)
Observed
Predicted

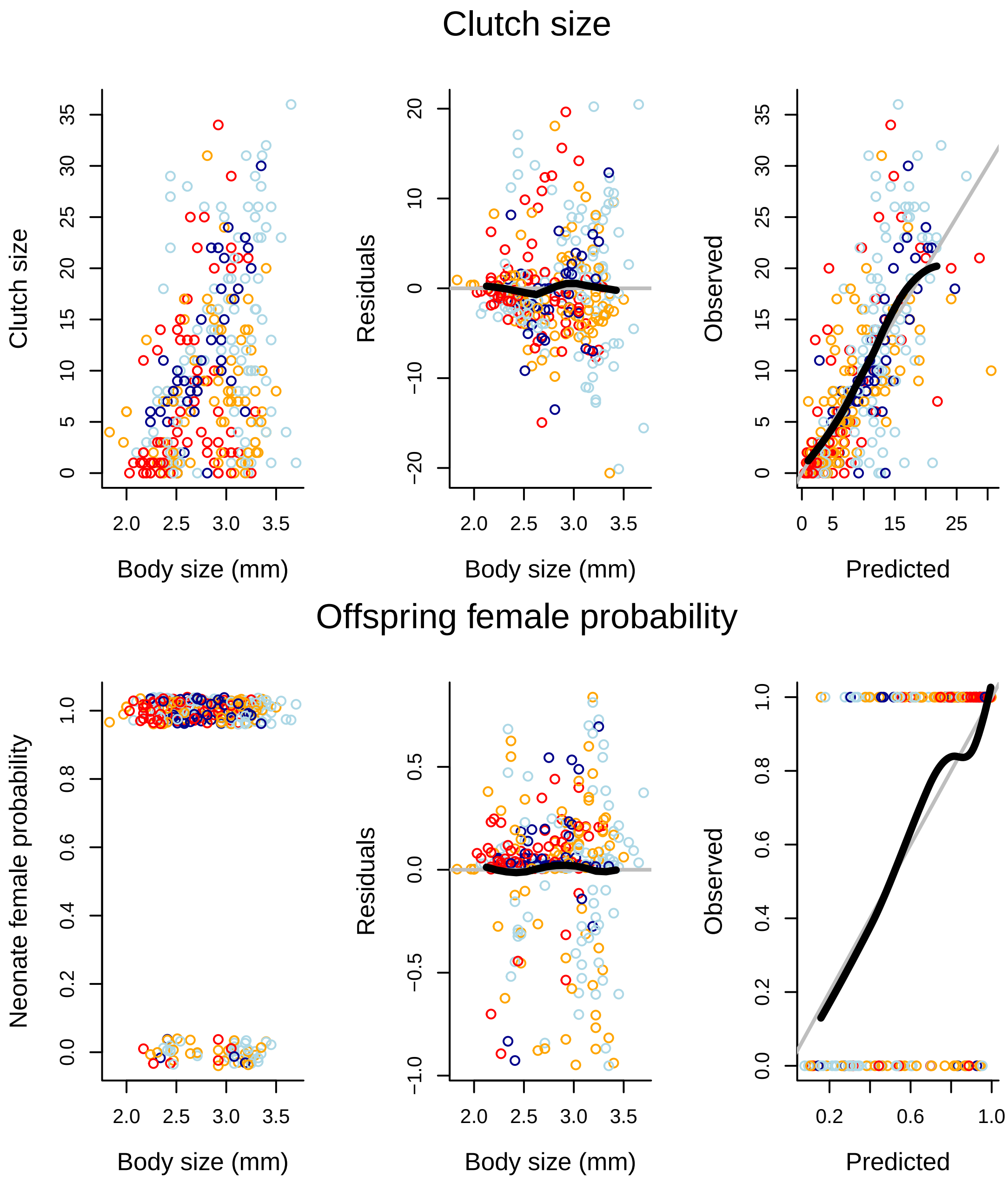

Clutch size
Clutch size
Body size (mm)
Residuals
Body size (mm)
Observed
Predicted
Offspring female probability
Neonate female probability
Body size (mm)
Residuals
Body size (mm)
Observed
Predicted

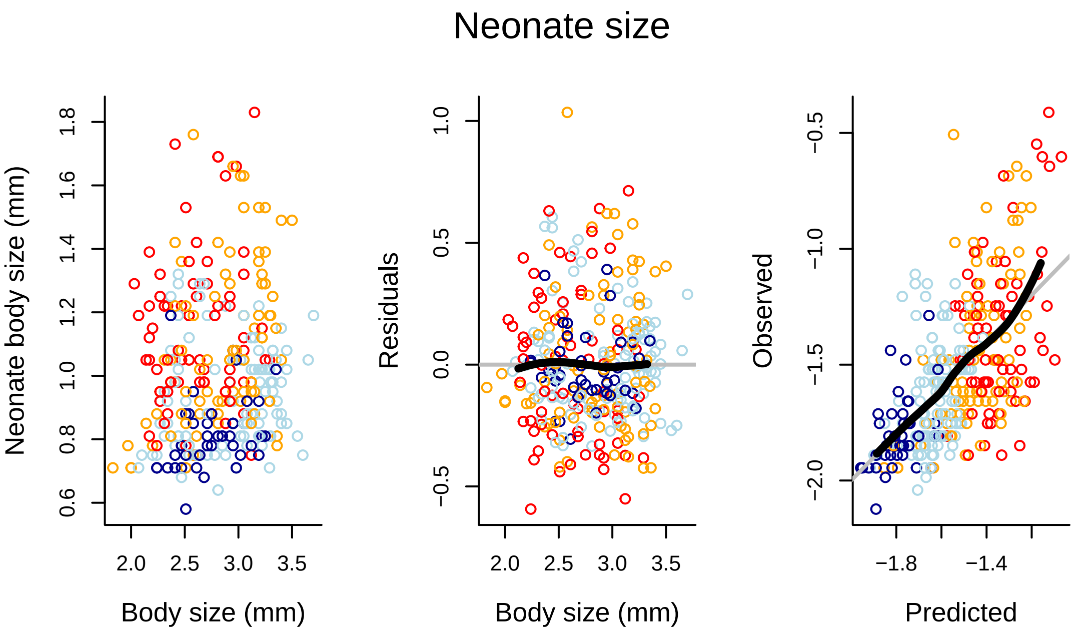

3 Variation among genotypes on competition outcomes

We used the estimated random effects of clonal identity, per vital rate model, to construct IPMs for specific genotypes (S_{1-6} and N_{1-6}), averaging genotype-specific vital rates across 1000 posterior draws. We then predicted coexistence outcomes for each pair of Northern-Southern genotypes, as described above. As a validation, we compared these equilibrium predictions to proportions from the fitted 'Observed genotype frequency model', which was directly informed by the observed genotype frequencies (Eq. 1 in the main text).

This analysis revealed considerable variation among genotypes in both their performance with respect to various vital rates and their overall competitiveness (Figure below). In line with previous results, lower temperatures generally favor Northern genotypes, and high temperatures favor Southern genotypes. Almost all clonal pairs are predicted to stably coexist for temperatures under 26 ^{o}C, but with an increasing temperature, Southern genotypes start outcompeting Northern genotypes in increasing numbers (colored dots). We compared these predictions to the observed genotype frequencies of each clonal combination (Eq. 1), validating that our model captures the observed shifts in genotype frequencies across all temperatures (correlation r=0.74; within each temperature: r ranges between 0.45-0.53).

As a measure of within-latitude competitiveness of each genotype, we averaged the equilibrium proportion of each genotype, when competing with genotypes from opposing latitudes, across all evaluated temperatures (panel C, symbols and colors match panel A-B). To assess which vital rates drive this variation in competitiveness, we compared estimated random genotype effects in each vital rate, to the competitiveness. Genotype-specific variation in survival show a strong correlation with competitiveness (r=0.94 and r=0.91 for Northern and Southern genotypes, respectively), indicating that within-latitude clonal variation in survival explains within-latitude competitive ability to a large extent (note that estimates are based on six data points only). Within-latitude variation in somatic growth, neonate release probability, and female probability show significant correlations among the genotypes from one latitude.

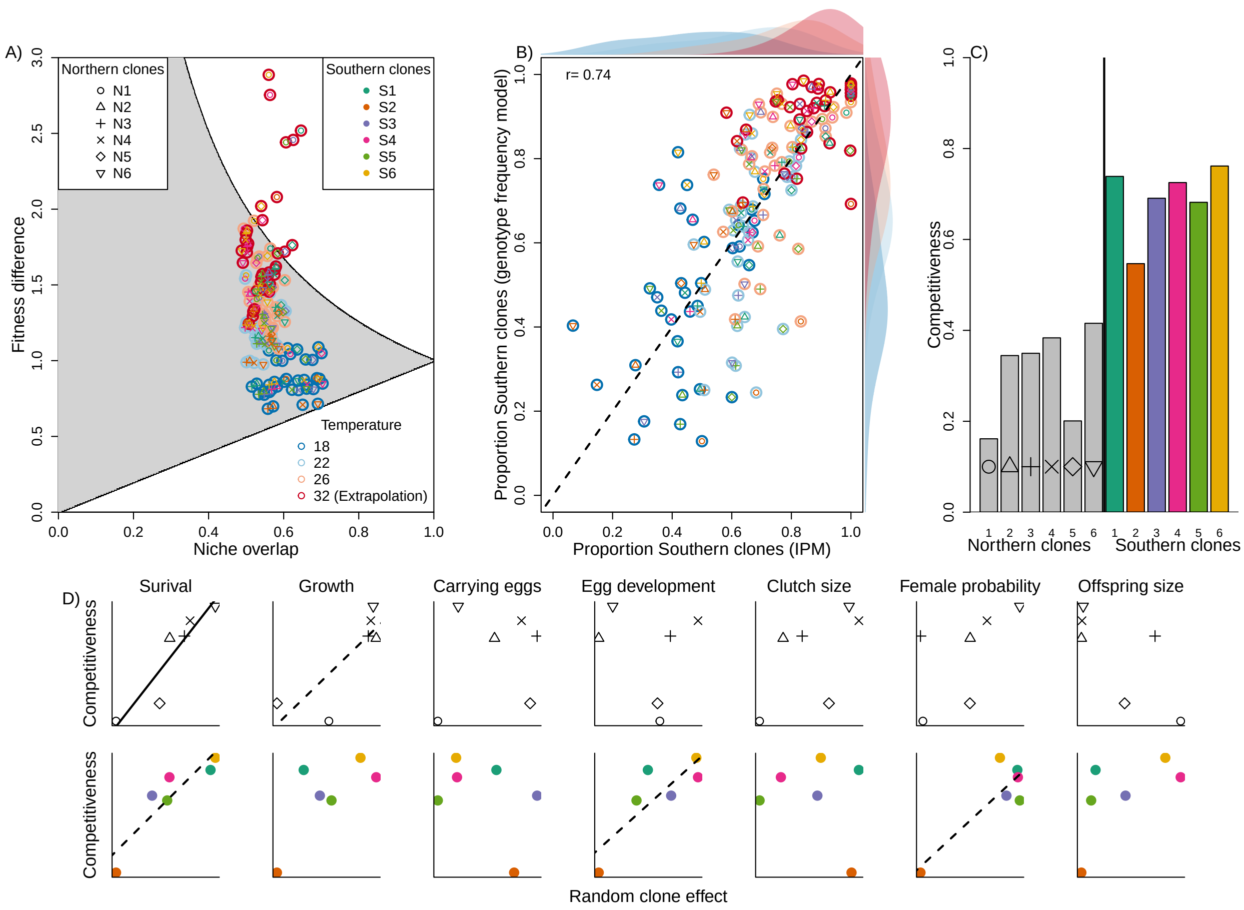

Figure 2: Variation among genotypes in competition outcomes and the contribution of underlying vital rates. A) Coexistence outcomes in terms of niche overlap and competitive ability difference for each Northern-Southern combination of genotypes, at different temperatures (colored circles). Northern genotypes are indicated by the symbols within these dots, and their color refers to the different Southern genotypes, see legend. B) Relationship between the predicted equilibrium proportion of Southern genotypes using the IPM, and the estimated proportion based on the observed genotype frequencies at the end of the experiment (29 days). Dotted line shows x-y line. Colors and symbols match panel A). Density distributions for each temperature (see legend in A)) are shown next to the right and upper side of the graph. C) Competitiveness of each genotype, calculated as the predicted average equilibrium proportion of each genotype across all Northern-Southern competition treatments and temperatures. Colors and symbols as in A). D) Competitiveness as calculated in C) on the y-axis, against the estimated random genotype effects (x-axis), depicted per vital rate (columns) and per latitude (rows). Solid lines show significant relationships at significance level alpha<0.01; dotted lines show significant relationships for alpha<0.05. Symbols and colors as in A).

4 Genotype frequency model

The genotype frequency model (see Eq. 1 in the manuscript), fitted to the observed clonal identity of individuals sampled from the experimental populations, revealed temperature-dependent competition outcomes. We found a positive day effect (at a temperature of 21 ^{o}C, where the normalized temperature value was 0) on the probability of sampling Southern clones, although 95% credible intervals overlapped with 0 ($\beta_1 = 0.02$ [-0.003, 0.04] 95% CI). At higher temperatures, the effect of day becomes significantly more pronounced ($\beta_2 = 0.03$ [0.002, 0.05]), indicating that the frequency of the Southern clones increases with day and with temperature (Figure below).

Recall that we use this regression model for two purposes: 1) For estimating sympatric and allopatric densities, by multiplying total densities by predicted proportions. 2) As a validation for our IPM predictions, comparing IPM predictions on equilibrium proportions of Southern or Northern clones, to the predicted proportions based on the genotype frequency model. See the manuscript for more details.

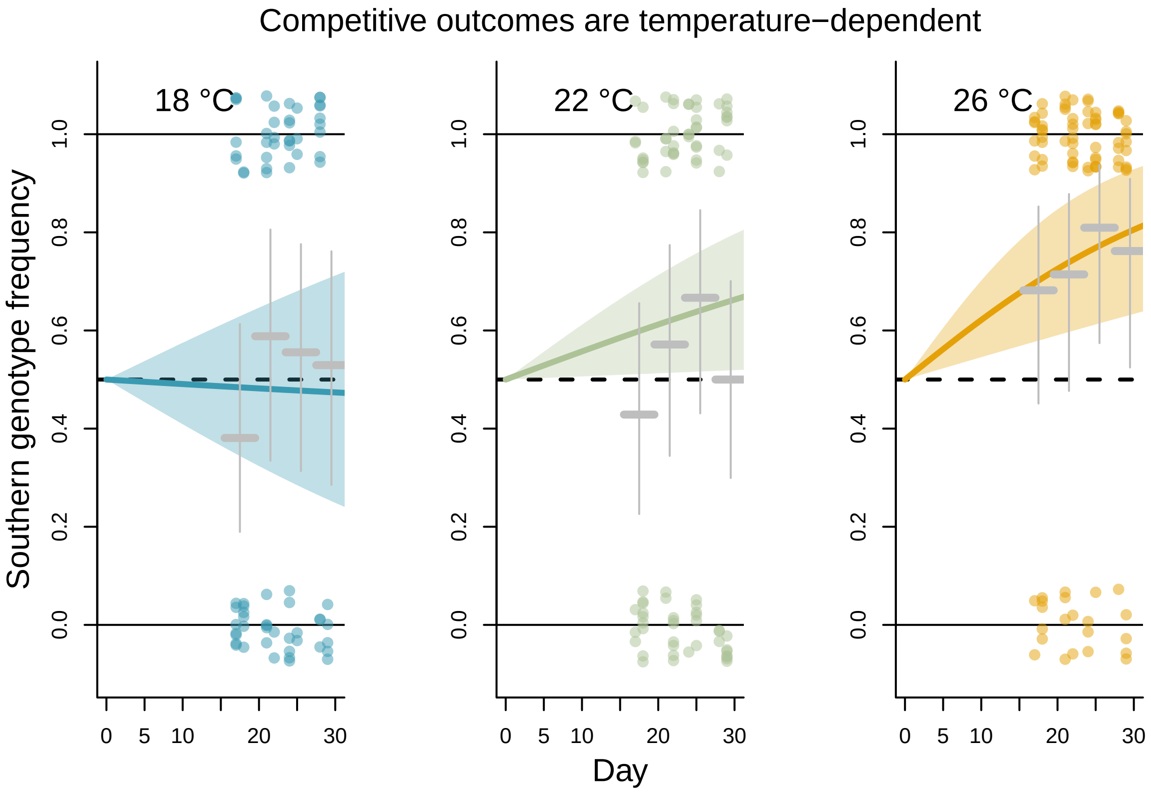

Figure 3: Competition outcomes between Southern and Northern clones are temperature-dependent. Dots show identified clones, where values of 0 (scattered around 0 to increase visibility) indicate identified Northern genotypes; values of 1 indicate identified Southern genotypes. Note that we only started sampling individuals for clonal identification after day 15. Lines show the predicted probability of sampling a Southern clone (i.e., measuring Southern clone frequency) based on a logistic mixed-effects model (Eq. 1 in the manuscript) (the 'Observed genotype frequency' model), for each temperature. Shaded polygons show 95% credible intervals of the posterior predictive distributions. Black horizontal lines show moving averages of the observations, with vertical lines indicating the 95% binomial confidence intervals for the average proportion

5 Population growth rates at low density

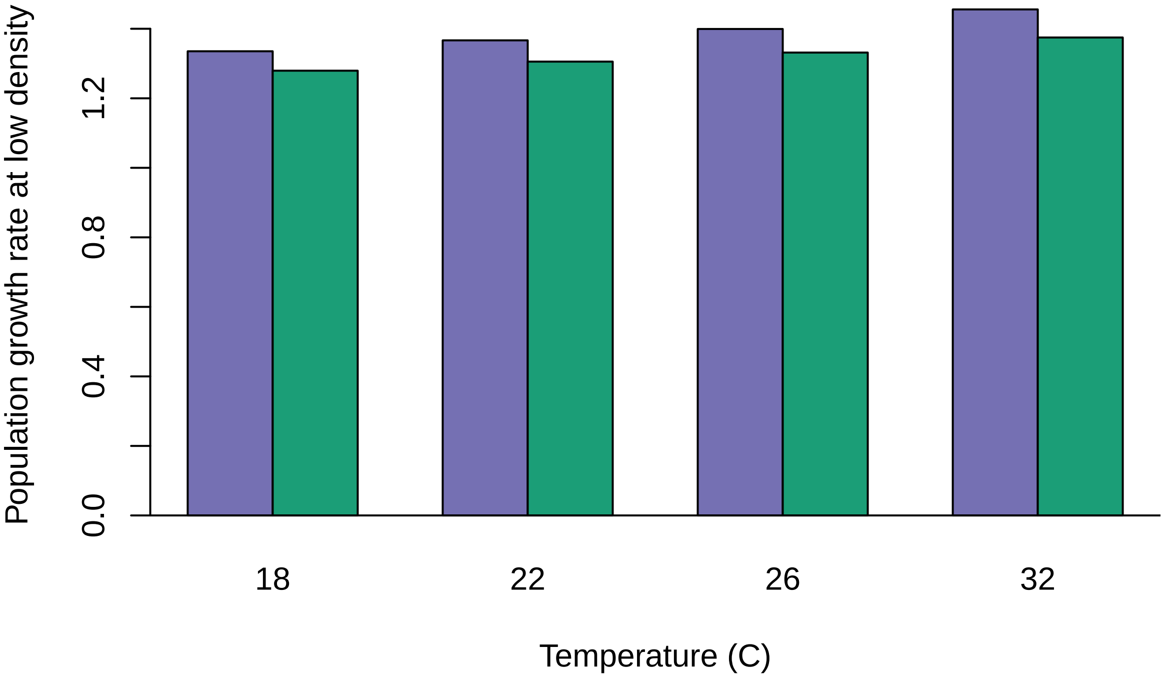

6 Produced ephippia per population

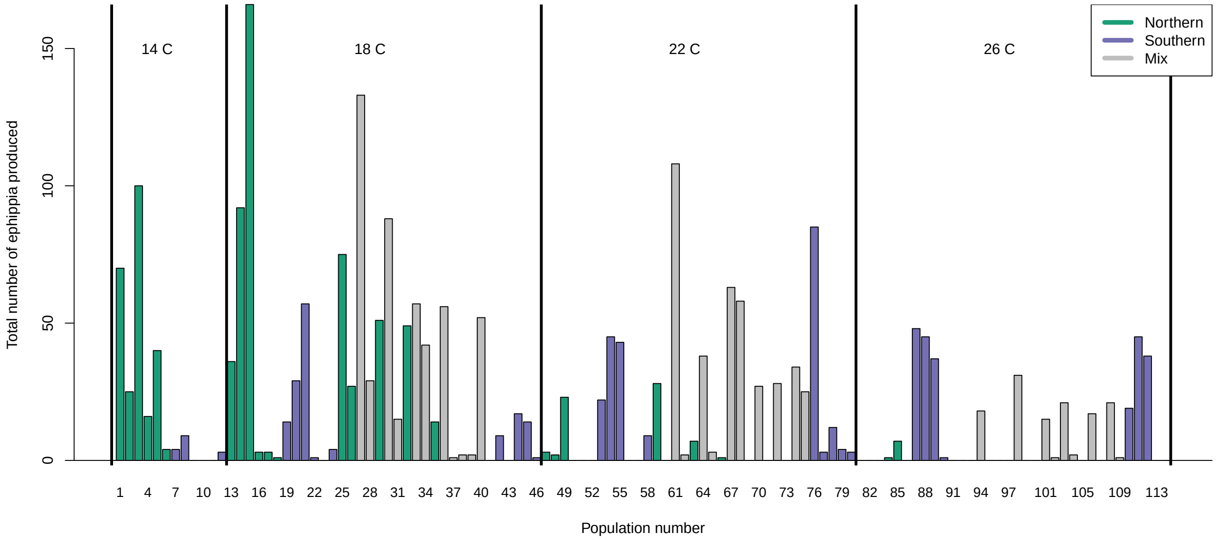

Supplement: Supplementary file 1 — Data S1: ele70214‐sup‐0001‐Supinfo.pdf. [file ELE-28-0-s001.pdf]
